# Bayesian modelling strategies for borrowing of information in randomised basket trials

**DOI:** 10.1111/rssc.12602

**Published:** 2022-10-28

**Authors:** Luke O. Ouma, Michael J. Grayling, James M. S. Wason, Haiyan Zheng

**Affiliations:** ^1^ Population Health Sciences Institute Newcastle University Newcastle upon Tyne UK; ^2^ MRC Biostatistics Unit University of Cambridge Cambridge UK

**Keywords:** biomarker‐guided trial, master protocol, personalised medicine, precision medicine, randomised controlled basket trial

## Abstract

Basket trials are an innovative precision medicine clinical trial design evaluating a single targeted therapy across multiple diseases that share a common characteristic. To date, most basket trials have been conducted in early‐phase oncology settings, for which several Bayesian methods permitting information sharing across subtrials have been proposed. With the increasing interest of implementing randomised basket trials, information borrowing could be exploited in two ways; considering the commensurability of either the treatment effects or the outcomes specific to each of the treatment groups between the subtrials. In this article, we extend a previous analysis model based on distributional discrepancy for borrowing over the subtrial treatment effects (‘treatment effect borrowing’, TEB) to borrowing over the subtrial groupwise responses (‘treatment response borrowing’, TRB). Simulation results demonstrate that both modelling strategies provide substantial gains over an approach with no borrowing. TRB outperforms TEB especially when subtrial sample sizes are small on all operational characteristics, while the latter has considerable gains in performance over TRB when subtrial sample sizes are large, or the treatment effects and groupwise mean responses are noticeably heterogeneous across subtrials. Further, we notice that TRB, and TEB can potentially lead to different conclusions in the analysis of real data.

## 1 INTRODUCTION

Improved understanding of molecular biology has facilitated rapid advances in identification of patient subgroups with better response to a targeted therapy. For instance, ado‐trastuzumab emtansine in *HER2* amplified ovarian and lung cancers (Li et al., 2018) and pembrolizumab in metastatic, microsatellite–instability–high cancer patients (Lemery et al., 2017) have demonstrated efficacy leading to FDA approval. Consequently, several innovative trial designs have emerged to efficiently evaluate targeted therapies under a single master protocol with the potential to expedite drug development (Woodcock & LaVange, 2017). The core idea in these designs is to (i) stratify patients with a given disease based on disease characteristics most predictive of treatment response and evaluate several linked targeted therapies (umbrella trials) (Siden et al., 2019; Woodcock & LaVange, 2017) or (ii) stratify patients with a shared potential therapeutic target in different underlying conditions based on their disease subgroups, evaluating a single therapy (basket trials) (Kaizer et al., 2019; Siden et al., 2019; Woodcock & LaVange, 2017). Trial designs (i) and (ii) can be randomised or non‐randomised and can allow addition of arms and incorporation of adaptive design considerations.

**FIGURE 1:**
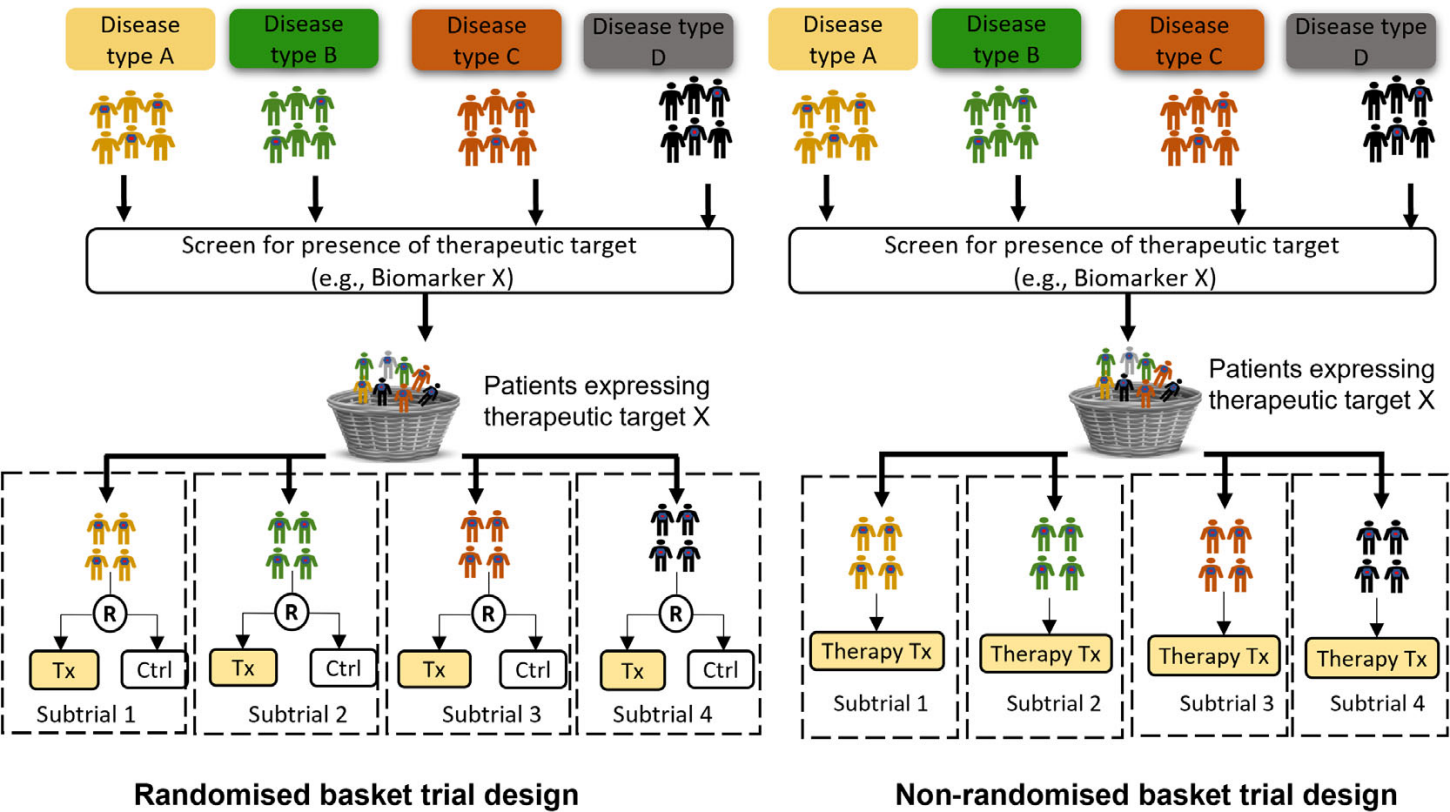
Illustration of the infrastructure of randomised and non‐randomised basket trial designs [Colour figure can be viewed at wileyonlinelibrary.com]

This paper will focus on basket trials that evaluate a single treatment for multiple disease indications with a common characteristic (Siden et al., 2019; Tao et al., 2018; Woodcock & LaVange, 2017), which could be a common genomic biomarker, mechanism of drug activity or clinical symptom that the treatment targets. Figure 1 illustrates randomised and non‐randomised basket trial designs, respectively. An example of a basket trial is that of TRK inhibitor Larotrectinib in 17 unique rare TRK fusion‐positive cancers (Drilon et al., 2018). This study enrolled both paediatric and adult patients and reported a 75% overall response rate, demonstrating the utility of basket designs in streamlining drug development in rare diseases.

In general, statistical analysis of basket trials follows one of three approaches: (i) stand‐alone analysis where subtrials are conducted and analysed separately as if they are individual studies (an example is the imatinib basket trial [Heinrich et al., 2008]); (ii) pooled analysis where all patients are considered as ‘homogenous’ and thus combined as one pooled data set, as in the basket trial of Larotrectinib (Drilon et al., 2018); or (iii) an analysis strategy that accounts for potential heterogeneity in treatment response across subtrials (Kaizer et al., 2019; Pohl et al., 2021). In practice, considerable heterogeneity in treatment response across subgroups within a basket trial can be observed. An example is the basket trial by Hyman et al. where vemurafenib displayed efficacy in BRAF‐V600‐mutant NSCLC and Erdheim‐Chester disease patients but not in colorectal cancer patients (Hyman et al., 2015). Consequently, there is a need to account for potential heterogeneity in both design and analysis; one basket trial accounting for heterogeneity is the ongoing Chinese Bayesian basket trial of Danzhu Fuyuan Granule as Adjunctive Therapy for Chronic Stable Angina, Vascular Dementia, and Idiopathic Membranous Nephropathy (NCT04498962).

Several statistical analysis methods accounting for potential heterogeneity (type [iii] analyses above) in basket trials have been proposed and/or implemented, using either Bayesian approaches, frequentist approaches or a combination of both. Such methods enable borrowing of information across subtrials; consequently, the statistical power may be increased for detecting that a treatment benefits a specific subgroup. This could be particularly important when a basket trial involves rare subgroups. Pohl et al. present a comprehensive review of existing statistical analysis methods that incorporate borrowing of information in basket trials (Pohl et al., 2021). Notable Bayesian methods include Bayesian hierarchical modelling (Berry et al., 2013), EXNEX (Neuenschwander et al., 2016), Simon's basket design (Simon et al., 2016), calibrated Bayesian hierarchical models (Chu & Yuan, 2018a), Bayesian latent subgroup design (Chu & Yuan, 2018b), Bayesian model averaging (Asano & Hirakawa, 2020; Psioda et al., 2019), Bayesian cluster hierarchical models (Chen & Lee, 2020), and the commensurate predictive prior (CPP) approach (Zheng & Wason, 2022). Notably, majority of these methods consider a single‐arm, non‐randomised trial setting that is often used in early‐phase oncology trials.

Recently, there has been increased interest in conducting randomised precision medicine trials, whenever possible, to improve the chances of a successful phase III trial (Saad et al., 2017), even in rare diseases (Lasch et al., 2017; Prasad & Oseran, 2015). The potential and growing use of basket trials outside of oncology carries even greater prospects for randomisation as an important feature in the design of future basket trials. Currently, randomised basket trials have been implemented in oncology (IMPACT II trial, NCT02152254), HIV/AIDS (Moore et al., 2021), autoimmune and neurological disorders like primary biliary cholangitis (PBC) and Parkinson's disease (Zheng & Wason, 2022), auto‐inflammatory syndromes like familial Mediterranean fever and TRAP syndrome (De Benedetti et al., 2018), and have been proposed for immune‐mediated inflammatory diseases (Grayling et al., 2021). Evidently, development of statistical methodology for randomised basket trials will be of great importance.

A key question to randomised basket trials relates to how borrowing of information is handled in the analysis. In general, approaches to borrowing information involve consideration of how similar the treatment response is across subtrials. In a two‐arm setting, however, this could be achieved by considering the treatment effects across subtrials (referred to as ‘Treatment Effect Borrowing’, TEB) or the responses per treatment arm (later called groupwise mean responses in this paper, and referred to as ‘Treatment Response Borrowing’, TRB) across subtrials. This raises two important issues. Firstly, there is a need for a suitable method for TRB to be proposed for randomised basket trials. Secondly, the question of which borrowing approach is better is important; it is plausible that either method might be best in different situations. It is these points that this paper seeks to address.

This paper builds on work by Zheng and Wason (2022), which proposed a CPP approach that models the commensurability of treatment effects to leverage information from complementary subtrials (i.e., it facilitates TEB). We propose a new modified implementation of their methodology that models the commensurability of the groupwise mean responses to permit borrowing of information across subtrials (i.e., we extend their approach to allow for TRB). The groupwise mean responses refer to the mean response in either the control or experimental treatment arm within a subtrial. It is expected that this new TRB approach could outperform the original TEB modelling strategy if the response in one arm (e.g., the experimental treatment) is more similar across subtrials than the other (e.g., the control). We then evaluate the new and existing Bayesian modelling strategies for borrowing of information in randomised basket trials under different scenarios, through a comprehensive simulation study.

Our research is motivated by a phase II randomised basket trial (hereafter referred to as the OACS trial) which evaluates the efficacy of Obeticholic acid for treating cognitive dysfunction in three distinct patient subgroups: early‐stage PBC, late‐stage PBC and Parkinson's disease. Each patient subgroup has a corresponding subtrial in which patients are randomised equally to a new targeted treatment or placebo. The primary outcome is the change in a composite score that measures the patients overall cognitive performance after 26 weeks of treatment, which is regarded as a continuous endpoint. Each subtrial is to be analysed separately; a secondary analysis will allow borrowing of information across subtrials. The exact model to be used, and whether TEB or TRB will be carried out, is currently not determined. This work is part of on‐going research to ascertain which approach will be used.

The remainder of this article is organised as follows. In Section 2 we elaborate on our proposed TRB approach, provide an overview of the TEB methodology, and describe the simulation study including considered scenarios and trial characteristics. We also provide a description of case studies that will be used to further investigate the relative merits of the methods considered. Section 3 presents our results and Section 4 a discussion.

## 2 METHODS

In this paper, we focus on the commensurate prior approach (Zheng & Wason, 2022) to implementing borrowing of information across patient subgroups in randomised basket trials based on two modelling strategies. To be more precise, we parameterise the level of commensurability in terms of (i) the subtrial‐specific treatment effects (TEB) or (ii) the mean responses by treatment groups across the subtrials (TRB). We elaborate on an existing commensurate prior approach incorporating TEB in Section 2.1 (Zheng & Wason, 2022), and develop a new strategy for TRB in Section 2.2. Both will be compared with stand‐alone analyses (i.e., no borrowing of information; referred to as ‘No Borrowing’, NB) in a comprehensive simulation study.

### 2.1 Treatment effect borrowing

Consider a basket trial that enrols patients in K subgroups. Furthermore, $n_{k}$ patients are randomised (equally) to receive a new treatment or a control per subtrial k=1,…,K. The primary endpoint in this basket trial is a continuous outcome, denoted by $y_{\mathrm{ik}}$, is collected from each patient $i=1,\ldots ,n_{k}$ in each subtrial k=1,….K. Let $\theta_{k}$ be the subtrial‐specific treatment effect and $T_{\mathrm{ik}}$ is a binary treatment assignment indicator; $T_{\mathrm{ik}}=1$ if patient i in subtrial k receives the experimental treatment and 0 otherwise (control). For simplicity, we consider here a regression model that includes just the intercept and treatment effect parameters

(1) $$y_{\mathrm{ik}}=\beta_{k}+\theta_{k}T_{\mathrm{ik}}+\varepsilon_{\mathrm{ik}}.$$

Then, for each subtrial k=1,….K, we have

$$E\left(y_{\mathrm{ik}}|T_{\mathrm{ik}}=1\right)=\beta_{k}+\theta_{k},\;E\left(y_{\mathrm{ik}}|T_{\mathrm{ik}}=0\right)=\beta_{k},$$

$i=1,..,n_{k}$, where $T_{\mathrm{ik}}$ denotes treatment allocation for the ith patient in subtrial k to either control ($T_{\mathrm{ik}}=0$) or the experimental treatment ($T_{\mathrm{ik}}=1$) and $\theta_{k}$ is the estimator of the experimental treatment benefit over control.

We use $k^{*}$ to label a specific subtrial of our local estimation interest. We wish to assess the degree of commensurability between $\theta_{k^{*}}$ and $\theta_{k}$, for $k\neq k^{*}$, to determine how much information we can leverage from complementary subtrial k. We introduce a precision parameter, denoted by $\upsilon_{kk^{*}}$, which captures the degree of consistency between the two location parameters $\theta_{k^{*}}$ and $\theta_{k}$. Then, $\theta_{k^{*}}$ would have a normal predictive prior centred on the complementary subtrial parameter $\theta_{k}$ such that

(2) θk*∣θk,υkk*∼Nθk,1υkk*2,k*≠k.

We place a spike and slab prior (Hobbs et al., 2012; Mitchell & Beauchamp, 1988) $g\left(\upsilon_{kk^{*}}\right)$ on each precision parameter $\upsilon_{kk^{*}}$. The spike and slab prior distribution is a discrete mixture distribution defined as locally uniform between two limits $0\leq \alpha_{l}\leq \alpha_{u}$ (the slab component), and with a probability mass concentrated at a point $S>\alpha_{u}$ (the spike component). Explicitly, the prior assumes that ω

(3) $$\begin{array}{ll} \mathbb{P}\left(\upsilon_{kk^{*}}<\alpha_{l}\right) & =0,\\ \;\mathbb{P}\left(\upsilon_{kk^{*}}<u\right) & =\omega_{kk^{*}}\left\{\frac{u-\alpha_{l}}{\alpha_{u}-\alpha_{l}}\right\},\alpha_{l}\leq u\leq \alpha_{u},\\ \;\;\mathbb{P}\left(\upsilon_{kk^{*}}>\alpha_{u}\right) & =\mathbb{P}\left(\upsilon_{kk^{*}}=S\right)=1-\omega_{kk^{*}}, \end{array}$$

where $\omega_{kk^{*}}$ denotes the prior probability that $\alpha_{l}\leq \upsilon_{kk^{*}}\leq \alpha_{u}$. A small $\omega_{kk^{*}}$ corresponds to a large value of $\upsilon_{kk^{*}}$ at *S*, thus allowing for strong borrowing of information. When the complementary subtrials are incommensurate, a greater probability mass is allocated to the slab prior through a large $\omega_{kk^{*}}$, hence information from the complementary subtrial is discounted.

To quantify $\omega_{kk^{*}}$, the prior belief about the incommensurability between subtrial k and $k^{*}$, we proceed by computing the distributional divergence between the posterior distributions $\pi_{k}\;\left(\theta_{k}|y_{k}\right)$ and $\pi_{k^{*}}\;\left(\theta_{k^{*}}|y_{k^{*}}\right)$ and relate these distances to the spike prior probability ($y_{k}$ and $y_{k^{*}}$ are outcome data from subtrial k and $k^{*}$, respectively, $k^{*}\neq k$). Following Zheng and Wason (2022), we use the Hellinger distance ($d_{H}$) to quantify this distributional divergence for three key reasons: (i) $d_{H}$ is a natural choice since $d_{H}\;\left(\pi_{\theta_{k^{*}}},\pi_{\theta_{k}}\right)\in \left[0,1\right]$ that is, it falls strictly in the desired interval such that $\omega_{kk^{*}}\in \left[0,1\right]$; (ii) $d_{H}$ is invariant to transformation; and (iii) symmetry, $d_{H}\;\left(\pi_{\theta_{k^{*}}},\pi_{\theta_{k}}\right)=d_{H}\;\left(\pi_{\theta_{k}},\pi_{\theta_{k^{*}}}\right)$. We compute $d_{H}$ as follows

(4) $$d_{H}\;\left(\pi_{\theta_{k^{*}}},\pi_{\theta_{k}}\right)=\sqrt{\frac{1}{2}\int_{-\infty }^{\infty }{\left(\sqrt{\frac{d\pi \left(\theta_{k^{*}}|y_{k^{*}}\right)}{d\theta }}-\sqrt{\frac{d\pi \left(\theta_{k}|y_{k}\right)}{d\theta }}\right)}^{2}d\theta }.$$

The prior probability $\omega_{{\mathrm{kk}}^{*}}$ can be defined as a function of the computed Hellinger distance, for the desire of incorporating commensurate information only. For simplicity, we let $\omega_{{\mathrm{kk}}^{*}}=d_{H}\left(\theta_{k^{*}},\theta_{k}\right)$.

As $d_{H}\;\left(\pi_{\theta_{k^{*}}},\pi_{\theta_{k}}\right)\to 1$, more probability mass is allocated to the slab prior, hence a smaller precision parameter (larger variance), which denotes a high degree of in commensurability between subtrials $k^{*}$ and k. Consequently, we down‐weight information from the corresponding complementary subtrial. On the contrary, as $d_{H}\;\left(\pi_{\theta_{k^{*}}},\pi_{\theta_{k}}\right)\to 0$, more probability mass is allocated to the spike prior, leading to a large precision parameter $\nu_{kk^{*}}$ (smaller variance). Consequently, information from the complementary subtrial is largely incorporated. The Hellinger distances are then normalised into a series of weights to determine how much information is leveraged from a complementary subtrial. When K≥3, the K−1 CPPs are combined into a marginal predictive prior for $\theta_{k^{*}}$.

The resulting marginal predictive prior for $\theta_{k^{*}}$ (Equation 5) is updated to the posterior using data from subtrial $k^{*}$ to inform decision‐making.

(5) $$\pi^{\mathrm{MPP}}\;\left(\theta_{k^{*}}|y_{k^{*}},y_{\left(-k^{*}\right)}\right)\propto L\left(y_{k^{*}}|\theta_{k^{*}}\right)\times \pi^{\mathrm{MPP}}\;\left(\theta_{k^{*}}|y_{\left(-k^{*}\right)}\right),$$

where π(·) and L(·) denote the prior/posterior distribution and likelihood function respectively. Specifically, *go* and *no‐go* decisions are made which refer to whether to recommend continuation or termination of investigation of the experimental therapy. Such decisions are made within all K subtrials, each testing the hypothesis that the experimental therapy provides benefit over control:

$$H_{0k}:\theta_{k}\leq 0\;\mathrm{vs}.\;H_{1k}:\theta_{k}>0,\;k=1,\ldots ,K.$$

Precisely, decisions are based on probabilistic inferences about $\theta_{k}$; a *go* decision is made if the estimated posterior probability that $\theta_{k}$ is greater than a prespecified threshold $\vartheta_{0}$ exceeds a desired probability level (e.g., 0.975), that is, $\mathbb{P}\left(\theta_{k}^{*}>\vartheta_{0}\;\right)>0.975$, otherwise a *no‐go* decision is taken. The threshold $\vartheta_{0}$ represents a nominated magnitude of improvement that provides compelling evidence of experimental treatment benefit over control.

This TEB approach accommodates (i) complete discarding of information if $\theta_{k}$ and $\theta_{k^{*}}$ are largely incommensurate and (ii) reduces to the complete pooling approach if the parameters are commensurate. In the special case when K=2, and there is no a priori assumption about which subtrial has a larger treatment effect, the degree of borrowing between $\theta_{1}\;\text{and}\;\theta_{2}$ can be anticipated to be symmetric (although this may differ) due to the symmetry property of the Hellinger distance, following (Equation 4) to define the spike and slab prior.

### 2.2 Treatment response borrowing

Let j=E,C index the experimental treatment and control groups, respectively. Denote the groupwise mean response by $\varphi_{\mathrm{jk}}$, for subtrial k=1,…,K. The groupwise mean response refers to the mean for a treatment group j within a subtrial k. Given that $\varphi_{jk^{*}}$ is our subtrial‐specific parameter of interest, we define subtrial $k^{*}\;$ and k $\left(k^{*}\neq k\right)$ as our contemporary and complementary subtrials, respectively. Let $y_{k}^{\left(j\right)}$ represent outcome measurement from treatment arm j in subtrial k. We place an operational prior $\pi_{0k^{*}}\left(\varphi_{jk^{*}}\right)$ on the parameter $\varphi_{jk^{*}}$. Using Bayes' Theorem, we have an operational posterior as follows

(6) $$\pi_{k^{*}}\;\left(\varphi_{jk^{*}}|y_{k^{*}}^{\left(j\right)}\right)\propto L\left(y_{k^{*}}^{\left(j\right)}|\varphi_{jk^{*}}\right)\pi_{0k^{*}}\;\left(\varphi_{jk^{*}}\right),$$

which represents information carried by the contemporary subtrial k*.

To facilitate information borrowing, we make use of a CPP—a normal predictive prior centred at $\varphi_{\mathrm{jk}}$, with a precision parameter $\nu_{kk^{*}}^{\left(j\right)}$. For treatment arm j, we can represent the normal predictive distribution for $\varphi_{jk^{*}}$ as

(7) φjk*∣φjk,νkk*(j)∼Nφjk,1νkk*(j)2.

As such, we have the CPP as

(8) $$\pi^{\mathrm{CPP}}\;\left(\varphi_{jk^{*}},\nu_{kk^{*}}^{\left(j\right)}|y_{k}^{\left(j\right)},\varphi_{\mathrm{jk}}\right)\propto L\left(y_{k}^{\left(j\right)}|\varphi_{\mathrm{jk}}\right)\pi_{0k}\left(\varphi_{\mathrm{jk}}\right)\times \nu_{kk^{*}}^{\left(j\right)}\varphi \left(\left(\varphi_{jk^{*}}-\varphi_{\mathrm{jk}}\right)\nu_{kk^{*}}^{\left(j\right)}\right)\times g\left(\nu_{kk^{*}}^{\left(j\right)}\right),$$

where $g\left(\nu_{kk^{*}}^{\left(j\right)}\right)$ denotes the prior distribution of the unknown parameter $\nu_{kk^{*}}^{\left(j\right)}$ and φ is the standard normal density function. The choice of $g\left(\nu_{kk^{*}}^{\left(j\right)}\right)$ is important to allow for robust borrowing on treatment arm j from subtrial k to $k^{*}$; specifically, we expect that (i) information can be completely discarded if $\varphi_{jk^{*}}$ and $\varphi_{\mathrm{jk}}$ are incommensurate, $\nu_{kk^{*}}^{\left(j\right)}\approx 0$, and (ii) strong borrowing occurs when subtrial $k^{*}$ and k are largely commensurate, $\nu_{kk^{*}}^{\left(j\right)}\gg 0$.

Our new modelling strategy allows for more flexible borrowing of information between subtrials, using their commensurability in either the experimental or control treatment, or both. Thus, for each precision parameter $\nu_{kk^{*}}^{\left(j\right)}$, we place a spike and slab prior with respective prior probabilities of $\left(1-\omega_{kk^{*}}^{\left(j\right)}\right)$ and $\omega_{kk^{*}}^{\left(j\right)}$:

$$\mathbb{P}\left(\nu_{kk^{*}}^{\left(j\right)}<\alpha_{l}\right)=0,$$

$$\mathbb{P}\left(\nu_{kk^{*}}^{\left(j\right)}<u\right)=\omega_{kk^{*}}^{\left(j\right)}\left\{\frac{u-\alpha_{l}}{\alpha_{u}-\alpha_{l}}\right\},\alpha_{l}\leq u\leq \alpha_{u},$$

(9) $$\mathbb{P}\left(\nu_{kk^{*}}^{\left(j\right)}>\alpha_{u}\right)=\mathbb{P}\left(\nu_{kk^{*}}^{\left(j\right)}=S\right)=1-\omega_{kk^{*}}^{\left(j\right)}.$$

Specifically, when $\omega_{kk^{*}}^{\left(j\right)}\to 0,$ $\nu_{kk^{*}}^{\left(j\right)}$ is as large as S≫0, thus allowing for strong borrowing of information.

To quantify $\omega_{kk^{*}}^{\left(j\right)}$, we compute the distributional divergence between the posterior distributions $\pi_{k^{*}}\left(\varphi_{jk^{*}}|y_{k^{*}}^{\left(j\right)}\right)$ and $\pi_{k}\left(\varphi_{\mathrm{jk}}|y_{k}^{\left(j\right)}\right)\;$ using the Hellinger distance discrepancy measure, $d_{H}$, (Equation 10) as previously described in Section 2.1 above.

(10) $$d_{H}\left(\pi_{\varphi_{\mathrm{jk}}},\pi_{\varphi_{{\mathrm{jk}}^{*}}}\;\right)=\sqrt{\frac{1}{2}\int_{-\infty }^{\infty }{\left(\sqrt{\frac{d\pi \left(\varphi_{{\mathrm{jk}}^{*}}|y_{k^{*}}^{\left(j\right)}\right)}{\mathrm{d\varphi}}}-\sqrt{\frac{d\pi \left(\varphi_{\mathrm{jk}}|y_{k}^{\left(j\right)}\right)}{\mathrm{d\varphi}}}\right)}^{2}\mathrm{d\varphi}}.$$

Likewise, we let $\omega_{kk^{*}}^{\left(j\right)}=d_{H}\left(\varphi_{\mathrm{jk}},\varphi_{{\mathrm{jk}}^{*}}\right)$ to incorporate information from the corresponding complementary subtrial as $d_{H}\left(\varphi_{\mathrm{jk}},\varphi_{{\mathrm{jk}}^{*}}\right)\to 0$ (high commensurability) and discount information when $d_{H}\left(\varphi_{\mathrm{jk}},\varphi_{{\mathrm{jk}}^{*}}\right)\to 1$ (high incommensurability).

In a randomised basket trial with K≥3 subtrials, we have K−1 complementary subtrials from which we may borrow information when estimating $\varphi_{{\mathrm{jk}}^{*}}$. From Equation (8), this translates to having K−1 CPPs marginally on $\varphi_{jk^{*}}$, hence we need to obtain a collective prior for $\varphi_{jk^{*}}$. In theory, we can see the location parameter estimate $\varphi_{jk^{*}}\;$ as a weighted sum of K−1 hypothetical random variables,

(11) $$\varphi_{jk^{*}}=\sum_{k=1,k\neq k^{*}}^{K}p_{kk^{*}}^{\left(j\right)}{\hat{\varphi }}_{\mathrm{jk}},\forall k^{*}=1,\ldots \;..,K,$$

where $p_{kk^{*}}^{\left(j\right)}$ quantifies the relative importance of information from arm j in subtrial k.

Given each of the K−1 CPPs as in Equation (8) can be represented approximately as a $N\left(\beta_{k}^{\left(j\right)},\tau_{k}^{2^{\left(j\right)}}\right)$ for ease of notation, we can suppose that ${\hat{\varphi }}_{\mathrm{jk}}∼N\left(\beta_{k}^{\left(j\right)},\tau_{k}^{2^{\left(j\right)}}\right)$. Then, the collective marginal predictive prior for $\varphi_{jk^{*}}$ would be as follows,

(12) $$\varphi_{jk^{*}}\;|\;y_{\left(-k^{*}\right)}∼N\left(\sum_{k=1,k\neq k^{*}}^{K}p_{kk^{*}}^{\left(j\right)}\beta_{k^{*}}^{\left(j\right)},\sum_{k=1,k\neq k^{*}}^{K}{{p_{kk^{*}}^{\left(j\right)}}^{2}\tau }_{k^{*}}^{2^{\left(j\right)}}\right),$$

To obtain $p_{kk^{*}}^{\left(j\right)}$ (the weight representing the degree of commensurability between $\varphi_{jk^{*}}$ and $\varphi_{\mathrm{jk}}$, $k\neq k^{*}$), we organise the pairwise discrepancy measurements into a symmetric K×Kmatrix

$$d_{H}\left(\pi_{\varphi_{\mathrm{jk}}},\pi_{\varphi_{{\mathrm{jk}}^{*}}}\right)=\left(\begin{array}{ccc} 0 & d_{12}^{\left(j\right)} & \ldots \ldots \\ \begin{array}{c} d_{21}^{\left(j\right)}\\ \vdots \end{array} & \begin{array}{c} 0\\ \vdots \end{array} & \ldots \ldots \\ d_{k1}^{\left(j\right)} & d_{k2}^{\left(j\right)} & \ldots \ldots \end{array}\begin{array}{c} \begin{array}{c} d_{1k}^{\left(j\right)}\\ d_{2k}^{\left(j\right)} \end{array}\\ \vdots \\ 0 \end{array}\right),$$

where each column lists the pairwise discrepancies between $\varphi_{{\mathrm{jk}}^{*}}$ and $\varphi_{\mathrm{jk}}$, for k* = 1, …, *K*. We then normalise the discrepancies columnwise. A decreasing function given by $p_{kk^{*}}^{\left(j\right)}=\frac{\exp\left(-d_{{\mathrm{kk}}^{*}}^{\left(j\right)}/R_{0}\right)}{\sum_{k}\exp\left(-d_{{\mathrm{kk}}^{*}}^{\left(j\right)}/R_{0}\right)}$, has been shown to have satisfactory properties to compute these weights (Zheng et al., 2020; Zheng & Wason, 2022). The largest weight is assigned to a subtrial with the smallest $d_{{\mathrm{kk}}^{*}}^{\left(j\right)}$ per treatment arm j. Here $d_{{\mathrm{kk}}^{*}}^{\left(j\right)}$ is the Hellinger distance when performing borrowing in treatment arm j across the k subgroups while $R_{0}$ controls the influence of the $d_{H}$ on the weights. The value $R_{0}$ of is pre‐defined here to be 0.15 as in Zheng and Wason (2022). Simulation results elsewhere have shown that small values are preferred as the methodology identifies commensurate subtrials more sensitively relative to when $R_{0}$ is large where it loses this ability (Zheng & Wason, 2022). When $R_{0}$ is sufficiently large ($R_{0}\gg d_{{\mathrm{kk}}^{*}}^{\left(j\right)}$), the weights will be approximately the same irrespective of the magnitude of the pairwise Hellinger distance. However as $R_{0}$ tends to 0 ($R_{0}\to 0^{+}$), then the weights $p_{kk^{*}}^{\left(j\right)}\to 1$ as $d_{{\mathrm{kk}}^{*}}^{\left(j\right)}\to 0$. Thereafter, we update the marginal predictive prior (Equation 12) using information from subtrial k,

(13) $$\pi^{\mathrm{MPP}}\left(\varphi_{jk^{*}}|y_{k^{*}}^{\left(j\right)},y_{\left(-k^{*}\right)}^{\left(j\right)}\right)\propto L\left(y_{k^{*}}^{\left(j\right)}|\varphi_{jk^{*}}\right)\times \pi^{\mathrm{MPP}}\left(\varphi_{jk^{*}}|y_{\left(-k^{*}\right)}^{\left(j\right)}\right),$$

where $y_{\left(-k^{*}\right)}^{\left(j\right)}$ denotes information from all subtrials except that from the contemporary subtrial $k^{*}$for arm j.

Although we implement borrowing by treatment response based on $\varphi_{jk^{*}}$, our interest lies on the subtrial‐specific treatment effect defined as $\theta_{k}=\varphi_{\mathrm{Ek}}-\varphi_{\mathrm{Ck}}$ for subtrial k. The decision‐making criteria (i.e., for *go* vs. *no‐go* conclusions) is the same as in Section 2.1 above based on $\theta_{k}$.

## 3 SIMULATION STUDY

### 3.1 Basic setting of the randomised basket trials

Consider a hypothetical phase II basket trial with K=5 subtrials. A maximum of 336 patients are to be recruited overall, and within each subtrial, k=1,…,5, unequal numbers of patients $\;n_{k}$ are to be enrolled: $n_{1}=70,n_{2}=66,n_{3}=64,n_{4}=n_{5}=68$. Our choice of sample sizes and number of subtrials is informed by recent basket trials. In particular, the CLUSTER randomised basket trial (De Benedetti et al., 2018) had 63–72 patients in two‐thirds of its subtrials, while several basket trials (Hyman et al., 2015; Slosberg et al., 2018) have examined a fairly large number of patient subgroups.

We simulate data from a hypothetical randomised basket trial as follows; we suppose that $y_{\mathrm{ik}}∼N\left(\mu_{\mathrm{ik}},\sigma^{2}\right)$ with $\mu_{\mathrm{ik}}=\beta_{0k}+\theta_{k}T_{\mathrm{ik}}$. We assume $\beta_{0k}=5$ and the inter‐patient SD σ=0.4. In the Bayesian analysis, we specify a random effects model for $\beta_{0k}$; $\beta_{0k}=\gamma_{0}+\varepsilon_{0}^{2}$. An uninformative normal prior is specified for $\gamma_{0}$, $\gamma_{0}∼N\left(0,5\right)$ and $\varepsilon_{0}^{2}∼\text{Half Normal}\left(1\right)$, where Half Normal(1) denotes a truncated N(0,1) to cover the range (0,∞).

Besides TRB and TEB, we also consider stand‐alone subtrial analyses, referred to as the NB approach. The NB approach implements a Bayesian analysis of the basket trial without sharing information across subtrials. Each of the subtrials is analysed in isolation. We set a normal prior $N\left(0,10^{2}\right)$ for each subtrial‐specific treatment $\theta_{k}$. If there is no information to be shared across subtrials, estimation of $\theta_{k}$ using the treatment effect and the difference in treatment responses is equivalent. Hence, we do not need to consider two versions of NB.

### 3.2 Simulation scenarios

We simulate randomised basket trial data under nine plausible scenarios listed in Table 1. We assume a known “true” treatment effect for each subtrial. Scenarios 1–4 feature the consistency of responses on both the experimental and control arms (not only the treatment effect) across subtrials. For scenarios 5 and 6, the set of treatment effects, that is, the values set for $\theta_{k}$, are generated from distinct multivariate normal distributions, with a high pairwise correlation coefficient (ρ=0.8) for low heterogeneity and a low pairwise correlation coefficient (ρ=0.1) for high heterogeneity, respectively. Furthermore, scenarios 5 and 6 feature the consistency of responses on the control arm only. Scenario 7 represents cases of inconsistency of responses on both experimental and control arms (not only the treatment effect) across subtrials. Scenario 8 is a case with consistent (null) treatment effects while scenario 9 has consistent responses on the control. Scenarios 1 and 8 are global null scenarios while scenario 9 is a mixed null scenario (some subtrials have non‐null treatment effect). The simulation parameters are chosen to represent plausible settings informed by varying specific parameters in recent basket trials with continuous endpoints.

**TABLE 1:** Description of simulation scenarios with specification of the ‘true’ treatment effect $\theta_{k}$ and mean response in the control group $\varphi_{\mathrm{Ck}}$

	Assumed treatment effect per subtrial (mean response in the control arm)					
Scenario	1	2	3	4	5	Description
Scenario 1	0 (5.0)	0 (5.0)	0 (5.0)	0 (5.0)	0 (5.0)	No treatment effect across all subtrials.
Scenario 2	0.35 (5.0)	0.35 (5.0)	0.35 (5.0)	0.35 (5.0)	0.35 (5.0)	Consistent $\theta_{k}^{\prime }s$ across all (small effect size) subtrials.
Scenario 3	0.55 (5.0)	0.55 (5.0)	0.55 (5.0)	0.55 (5.0)	1.20 (5.0)	$\theta_{k}^{\prime }s$ are consistent in all except subtrial 5, where the true effect is higher; mean response in control is the same.
Scenario 4	0.80 (5.0)	0.80 (5.0)	0.80 (5.0)	0.80 (5.0)	0.80 (10.0)	Consistent $\theta_{k}$ (large effect size) across subtrials, but subgroup 5 has larger mean response in both experimental and control arms.
Scenario 5	1.32 (5.0)	0.72 (5.0)	0.66 (5.0)	0.44 (5.0)	0.72 (5.0)	High heterogeneity in $\theta_{k}^{\prime }s$ across all subtrials (ρ=0.1).
Scenario 6	0.73 (5.0)	1.07 (5.0)	0.73 (5.0)	0.46 (5.0)	1.16 (5.0)	Low heterogeneity in $\theta_{k}^{\prime }s$ across subtrials (ρ=0.8).
Scenario 7	1.32 (3.5)	0.72 (5.0)	0.66 (4.2)	0.41 (6.5)	0.72 (5.6)	Same as scenario 5 except the mean response of control varies across subtrials.
Scenario 8	0 (2.5)	0 (4.0)	0 (5.5)	0 (7.0)	0 (8.5)	Same as scenario 1 except that the mean responses differ across subtrials.
Scenario 9	0 (5.0)	0 (5.0)	0.15 (5.0)	0.87 (5.0)	1.86 (5.0)	A mixture scenario of null, small, and large treatment effects.

We additionally evaluate the performance of the borrowing of information approaches for settings with smaller ($n_{S1}=12,n_{S2}=18,n_{S3}=10,n_{S4}=16,n_{S5}=14$) and larger ($n_{L1}=n_{L2}=100,n_{L3}=106,n_{L4}=110,n_{L5}=104$) subtrial sample sizes.

### 3.3 Performance evaluation metrics

We investigate the performance of the modelling approaches, focusing on the bias, mean square error (MSE), empirical standard error (EmpSE), coverage and width of the 95% posterior credible interval for $\theta_{k}$, and the Monte‐Carlo standard error (MCSE). These are given by

$$\text{Bias}\left(\theta_{k}\right)\approx \frac{1}{b_{\mathrm{sim}}}\sum_{l=1}^{b_{\mathrm{sim}}}{\hat{\theta }}_{k}^{l}-\theta_{k},$$

$$\mathrm{MSE}\left(\theta_{k}\right)\approx \frac{1}{b_{\mathrm{sim}}}\sum_{l=1}^{b_{\mathrm{sim}}}{\left({\hat{\theta }}_{k}^{l}-\theta_{k}\right)}^{2},$$

$$\text{EmpSE}\left(\theta_{k}\right)=\sqrt{\frac{1}{b_{\mathrm{sim}}-1}\sum_{l=1}^{b_{\mathrm{sim}}}{\left({\hat{\theta }}_{k}^{l}-{\bar{\theta }}_{k}\right)}^{2}},$$

$$\text{Coverage}\left(\theta_{k}\right)=\mathbb{P}\left({\hat{\theta }}_{k,\mathrm{low}}\leq \theta_{k}\leq {\hat{\theta }}_{k,\mathrm{upp}}\right)\approx \frac{1}{b_{\mathrm{sim}}}\sum_{l=1}^{b_{\mathrm{sim}}}1\left({\hat{\theta }}_{k,\mathrm{low}}^{l}\leq \theta_{k}\leq {\hat{\theta }}_{k,\mathrm{upp}}^{l}\right),$$

Width of the 95% credible interval, $\ell =\frac{1}{b_{\mathrm{sim}}}\sum_{l=1}^{b_{\mathrm{sim}}}\left(Q_{\theta_{k}}^{l}\left(0.975\right)-Q_{\theta_{k}}^{l}\left(0.025\right)\right)$,

$$\text{MCSE}\left(\theta_{k}\right)\approx \sqrt{\frac{1}{b_{\mathrm{sim}}\left(b_{\mathrm{sim}}-1\right)}\sum_{l=1}^{b_{\mathrm{sim}}}{\left({\hat{\theta }}_{k}^{l}-{\bar{\theta }}_{k}\right)}^{2}},$$

where $l=1,\ldots .,b_{\mathrm{sim}}\;$ indexes the simulated basket trials (i.e., $b_{\mathrm{sim}}$ is the total number of replicates performed for each simulation scenario); $\theta_{k}$ in the above is the assumed value in the particular simulation scenario; ${\hat{\theta }}_{k}^{l}$ is the estimate from one of the analysis models in Section 2 from the lth simulation replicate; ${\bar{\theta }}_{k}=b_{\mathrm{sim}}^{-1}\sum_{l}{\hat{\theta }}_{k}^{l}$ is the mean of the ${\hat{\theta }}_{k}^{l}$ across all replicates; and $Q_{\theta }^{l}\left(a\right)$, with 0 < *a* ≤ 100, denotes the *a*th quantile of the posterior distribution for *θ*; the lower and upper limits of the coverage definition are the 2.5th and 97.5th percentiles.

For each subtrial k, we also obtain the estimated posterior probability that $\theta_{k}$ exceeds the threshold $\vartheta_{0}\;$ to estimate type I error‐rates and power, $\mathbb{P}\left(\theta_{k}>\vartheta_{0}\;\right)>0.975$. For illustration, we specify $\vartheta_{0}=0.3$. Of interest is the subtrial‐wise and familywise type I error‐rates and the power for identifying effective subgroups. The subtrial‐wise type I error‐rate is the proportion of trials, with respect to a given subtrial, with an erroneous go decision made for a given null scenario (scenario 1). The familywise error‐rate is computed as the proportion of trials in which an incorrect go decision is made for any null hypothesis that is true. A similar approach to type I error‐rate and power computation has been used previously (Hobbs & Landin, 2018; Zheng & Wason, 2022).

We simulate 10,000 randomised basket trials for each of the simulation scenarios. For each replicate, we use two parallel chains each with 10,000 MCMC iterations. The first 3000 samples in each chain are discarded as burn‐in. Convergence of the MCMC chains was checked using Gelman‐Rubin diagnostic plots. All Bayesian analysis models are implemented in R (version 3.6.3) using the Rjags package. Both R and JAGS functions to reproduce the simulation study are available on GitHub (https://github.com/oondijo/RandBasketTrials).

### 3.4 Results

**FIGURE 2:**
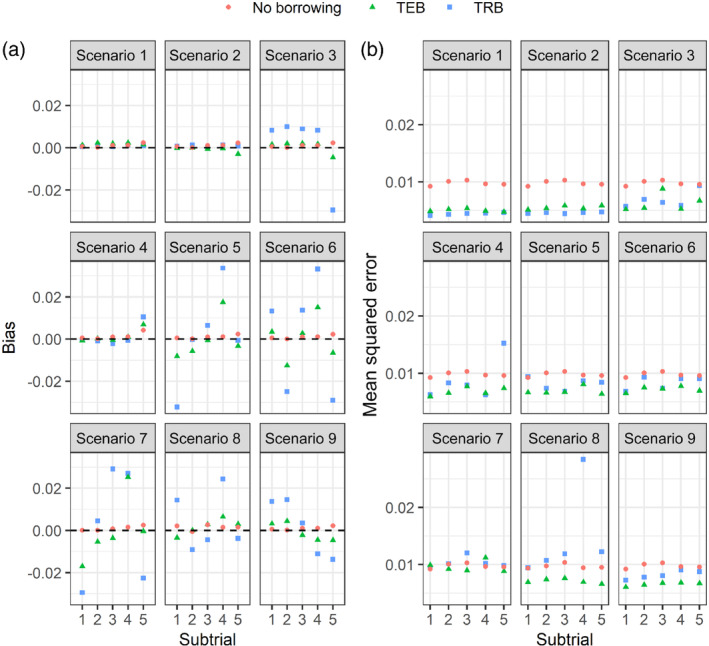
Comparison of three Bayesian analysis models for randomised basket trials, in terms of the bias and mean squared error of the estimators [Colour figure can be viewed at wileyonlinelibrary.com]

Figure 2 compares the TRB, TEB and NB analysis strategies in terms of the bias and MSE of the posterior mean estimators for the subtrial‐specific treatment effects. Under the global null scenarios, there is similar and noticeably small bias across the three approaches although TRB and TEB have smaller MSE especially in scenario 1. In scenario 8 (same as scenario 1 except that mean response across control, $\varphi_{\mathrm{Ck}}$, is different across subtrials), the bias and MSE for TEB and TRB are slightly different in some scenarios, occasionally higher for TRB. The MSEs of the point estimates differ considerably; the NB approach produces the highest MSE in scenarios 1–3 and comparable MSE to the TRB approach in scenarios 7–9.

When all or most subtrials are consistent in the mean responses on both the experimental and control arms (scenarios 1–4), the TRB and TEB approaches have comparable bias, but the TRB modelling approach has smaller. An exception to this is scenario 3, where all ${\varphi_{\mathrm{Ck}}}^{\prime }s$ and $\theta_{1}-\theta_{4}$ are similar, but $\theta_{5}$ is different; we observe here that TRB has slightly higher bias although the MSE are similar. In the mixed null scenario (scenario 9) where we anticipate very limited borrowing or heavy discounting of information, TEB produces smaller bias (similar to the NB approach) than TRB as well as the smallest MSE. We observe similar results under varying degrees of the treatment effect across the subtrials (scenarios 5 and 6). Overall, the TRB approach consistently provides smaller bias and MSE when the $\theta_{k}$ are small and similar across subtrials where response in control and experimental is also similar (for instance scenarios 1, 2 and 4), while TEB provides more accurate estimates when at least one of the true treatment effects and groupwise mean responses ($\varphi_{\mathrm{Ck}}$) are different (for instance scenarios 3, 8 and 9). In Figure S3, we demonstrate that these differences hold for the considered circumstances of smaller and larger basket trial sample sizes.

**FIGURE 3:**
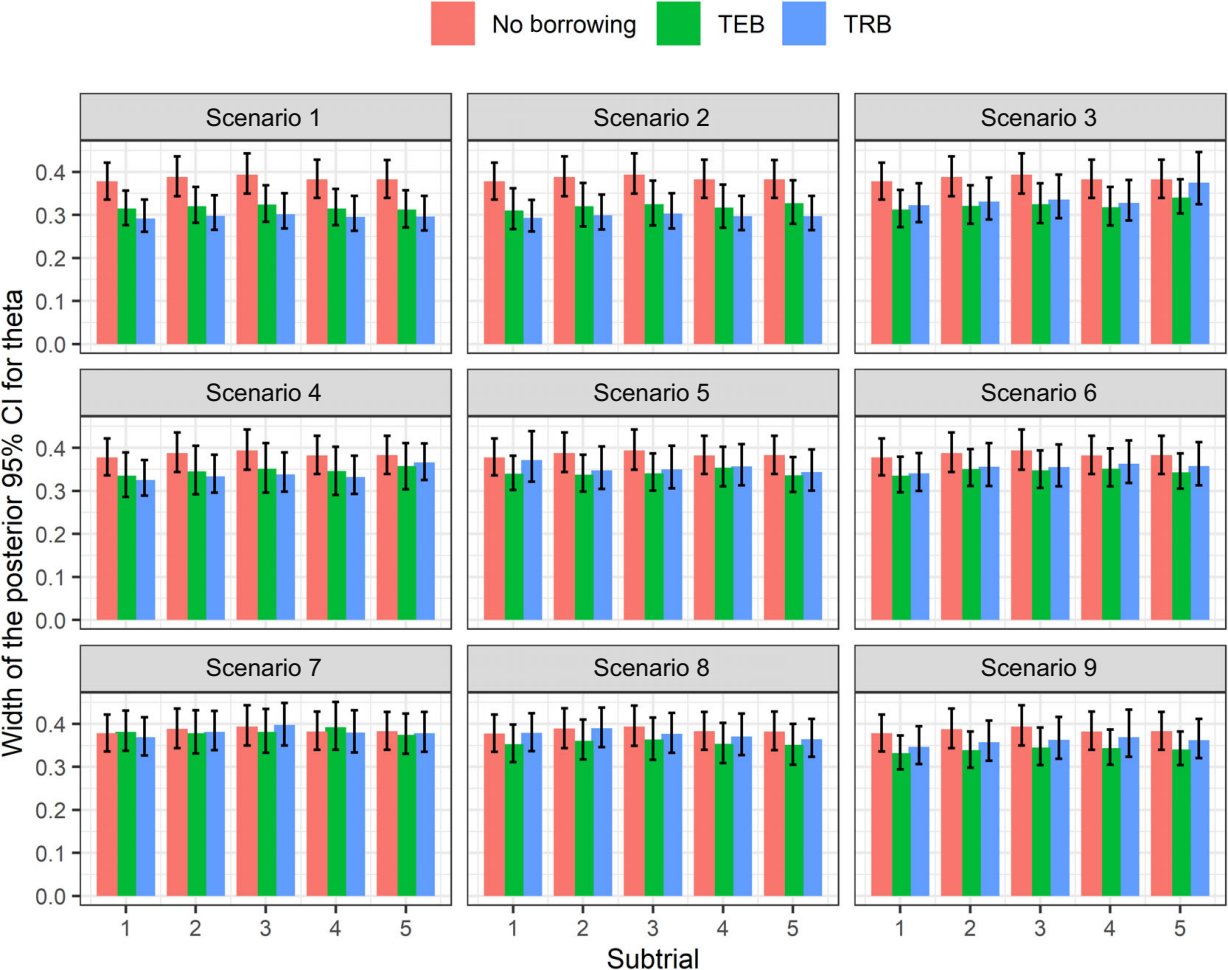
The median width of the 95% credible interval for posterior estimates $\theta_{k}$ obtained from different Bayesian analysis models. Error bars represent the 10th and 90th percentiles. [Colour figure can be viewed at wileyonlinelibrary.com]

Figure 3 visualises the median width of the posterior credible intervals for the $\theta_{k}$ yielded by the respective Bayesian analysis models. In all scenarios, we see that either TEB or TRB have the narrowest 95% credible intervals compared to the NB approach. The differences are particularly noticeable in scenarios 1–4 (apart from subtrial 5 of scenario 3 where differences are marginal) where treatment effects are consistent across all subtrials. This is because borrowing of information leads to greater efficiency gains and more precise posterior estimates of the treatment effect when subtrials are more commensurate. Although there are no observable differences in scenario 7 where both $\varphi_{\mathrm{Ck}}$ and $\theta_{k}$ are different across subtrials, it clear from Figure S6 that TRB works best under small sample sizes. However, in this scenario there are no differences across when the sample size is large, another indication that multiple metrics are necessary to distinguish TRB and TEB in that case.

For the global null scenario 1, we notice that TRB leads to more precise estimates, due to commensurability of mean responses of the control arm across all subtrials. However, when the mean response in control is different across subtrials (scenario 8), TEB gives the smallest posterior credible intervals across all subtrials. It is interesting to note that in circumstances of a small overall basket trial sample size, TRB gives the narrowest 95% credible intervals across nearly all scenarios; but for circumstances of a large sample size, TEB gives far narrower posterior credible intervals for the treatment effect in nearly all scenarios (Figure S6).

**FIGURE 4:**
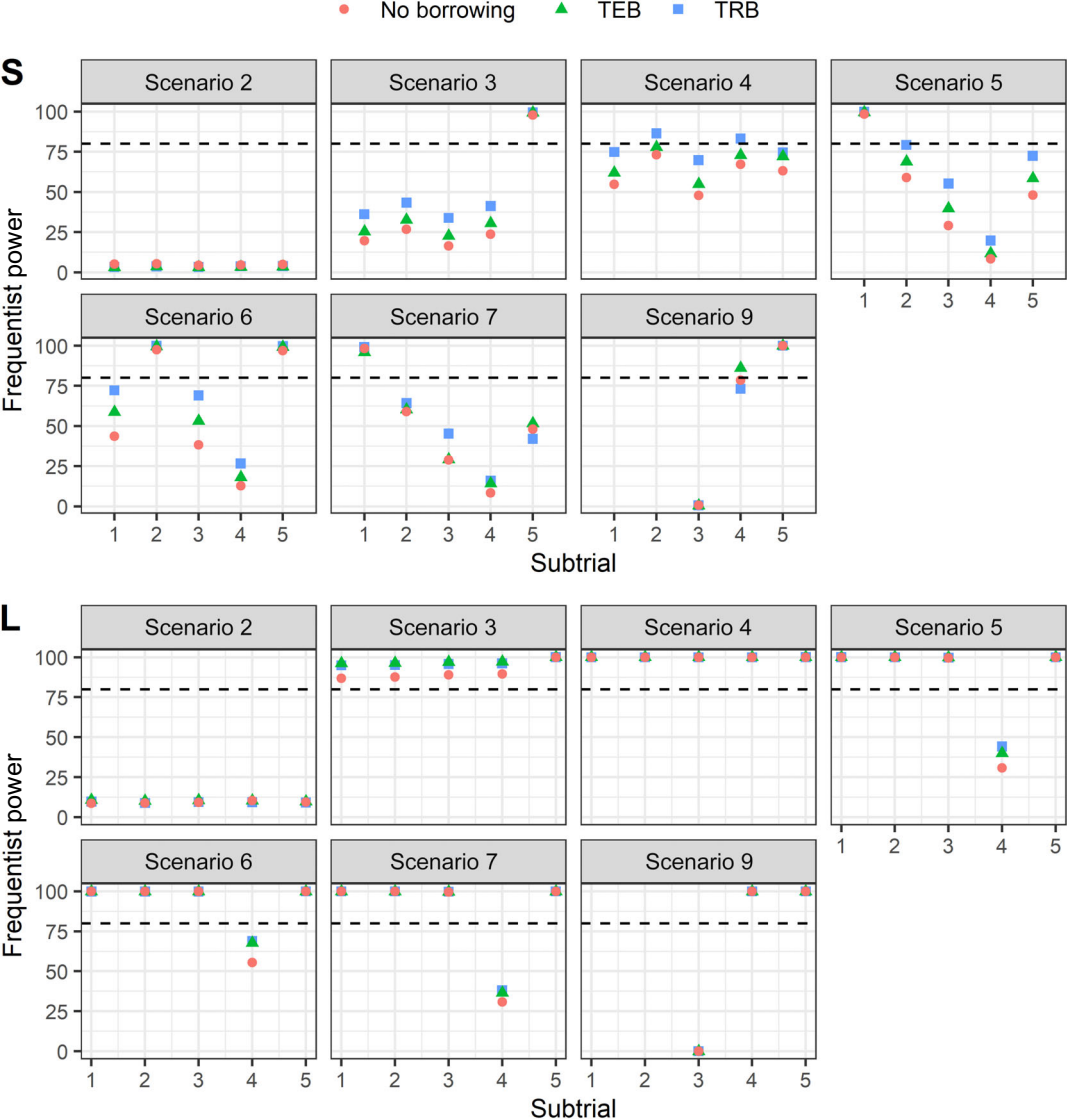
A comparison of the Bayesian analysis models for randomised basket trials, in terms of the frequentist power, under small (*S* – $n_{S1}=12,n_{S2}=18,n_{S3}=10,n_{S4}=16,n_{S5}=14$) or large (*L* – $n_{L1}=n_{L2}=100,n_{L3}=106,n_{L4}=110,n_{L5}=104$) sample sizes. Frequentist power is defined as the proportion of trials with a correct *go* decision under $H_{1k}:\theta_{k}>\vartheta_{0}$. Horizontal dashed line represents 80% power. [Colour figure can be viewed at wileyonlinelibrary.com]

We also compared the two modelling strategies for borrowing of information based on their statistical power, type I error‐rate, and familywise error‐rate. Figure 4 compares the statistical power under small (S) or large (L) sample sizes. The first thing to note is the increase in statistical power to demonstrate a treatment benefit in a particular subtrial as we leverage information from other subtrials. Both TRB and TEB, achieve higher statistical power in almost all scenarios compared to the NB approach.

We observe very low statistical power in scenario 2 ($\theta_{k}=0.35$ for all k), which is expected because *go* decisions based on $\mathbb{P}\left(\theta_{k}>0.3\right)>0.975$ are made to be very unlikely. In scenarios 3 and 4, where treatment effects are commensurate across most subtrials (except subtrial 5), we observe that TRB leads to higher statistical power over TEB and no borrowing. As the treatment effect gets larger (scenario 4), there is an increase in power, apart from subtrial 5 which is different in scenarios 3 and 4. Notably, the differences between the borrowing approaches are more pronounced when the subtrial sample sizes are small. For instance, in scenarios 3 and 4, TRB gives 0.3%–11% and 2%–15% higher power than TEB on average in subtrials 1–4 when sample sizes are small. However, when the sample size is larger, TEB outperforms TRB marginally (<2%) in nearly all scenarios. For the mixed global null (scenario 9), TEB yields at least 13% higher power than TRB in subtrial 4. Scenarios 5 and 7 mimic situations where the true $\theta_{k}$ are highly heterogeneous across all subtrials. Scenario 7 is further featured by the mean responses of the control group differing across the subtrials. We observe that TRB achieves the highest statistical power in scenario 5, but this is not always the case in scenario 7. In subtrial 5, TRB outperforms TEB by an increase of 14% in scenario 5, while in scenario 7 the gap is 10% in the opposite direction' (Figure 4‐S).

Principally, TRB achieves higher power compared to TEB when there is high commensurability across all subtrials, the mean response in control is similar across subtrials and the basket trial sample size is small. However, when either the treatment effects, and/or the mean response in the control/experimental arm are inconsistent across subtrials (scenarios 5–7 and 9), TRB still outperforms TEB in small sample sizes; in scenario 7 (subtrial 5) and scenario 9 (subtrial 4), TEB has higher power by at least 10% and 13%, respectively. When the sample size is larger, we notice TEB has marginal absolute gain in power in all scenarios over TRB. The observation that the power is high and similar across the three approaches in Figure 4 (bottom panel) is entirely expected. This is because when a treatment truly works, higher power will be observed as sample size gets larger for the same specified effect sizes. The low power in subtrial 4 in scenarios 5, 6 and 7 is because $\theta_{4}$ is assumed to be small (see Table 1) hence $\mathrm{Pr}\left(\theta_{k}>\vartheta_{0}\right)>0.975$ is expected to be low.

Table 2 quantifies the coverage probability of the posterior distributions based on the respective Bayesian models, as we vary the sample size. Both TEB and TRB approaches guarantee high coverage that is well above the target coverage (95%) in most scenarios. However, we notice substantial differences in the coverage for cases of small subtrial sample sizes. For example, in the global null scenario where the mean response in control is different across subtrials (scenario 8), TEB gives higher coverage for $\theta_{k}$ (which is also above the target) relative to TRB (approximately 4% in some cases). When the sample size is large, similar conclusions apply except that we observe smaller differences of 1%–2%. Further, TEB and TRB have similar coverage probability for $\theta_{k}$ (differences of <1%) in the null scenario 1 where the mean response in the control arm was assumed to be similar across subtrials. When treatment effects are assumed to be heterogeneous across subtrials (with subtrials inconsistent in the mean responses of the experimental or control arm, or both), we observe mixed results where TRB can perform poorest of the three (subtrial 1 of scenarios 5 and 7).

**TABLE 2:** Comparison of the coverage probability for the borrowing of information approaches for a Bayesian analysis of a randomised basket trial

		Small sample size					Large sample size			
		Subtrial					Subtrial			
Scenario	Borrowing approach	1	2	3	4	5	1	2	3	4	5
1	TRB	0.9706	0.9696	0.9708	0.9676	0.9667	0.9757	0.9798	0.9743	0.9764	0.9752
	TEB	0.9755	0.9776	0.9692	0.9731	0.9711	0.9716	0.9749	0.9721	0.974	0.978
	NB	0.9342	0.9422	0.9411	0.9438	0.9375	0.9505	0.9492	0.9471	0.9442	0.9526
2	TRB	0.9710	0.9694	0.9712	0.9683	0.9669	0.9770	0.9791	0.974	0.9737	0.9753
	TEB	0.9587	0.9586	0.9561	0.9567	0.9524	0.9718	0.9733	0.9709	0.9714	0.9689
	NB	0.9341	0.9421	0.9410	0.9438	0.9374	0.9505	0.9492	0.9471	0.9442	0.9526
3	TRB	0.9655	0.9646	0.9660	0.9626	0.9212	0.9658	0.9700	0.9644	0.9665	0.9515
	TEB	0.9575	0.9586	0.9558	0.9553	0.9384	0.9714	0.9726	0.9693	0.9695	0.9627
	NB	0.9340	0.9421	0.9411	0.9439	0.9373	0.9505	0.9493	0.9471	0.9442	0.9525
4	TRB	0.9680	0.9697	0.9671	0.9634	0.9482	0.9695	0.9721	0.9690	0.9669	0.9574
	TEB	0.9596	0.9627	0.9579	0.9557	0.9511	0.9672	0.9699	0.9679	0.9671	0.9623
	NB	0.9340	0.9421	0.9410	0.9439	0.9379	0.9505	0.9493	0.9471	0.9442	0.9521
5	TRB	0.9107	0.9620	0.9622	0.9303	0.9603	0.9430	0.9640	0.9603	0.9419	0.9621
	TEB	0.9359	0.9585	0.9546	0.9372	0.9519	0.9577	0.9645	0.9608	0.9504	0.9649
	NB	0.9341	0.9421	0.9410	0.9438	0.9374	0.9504	0.9493	0.9471	0.9442	0.9525
6	TRB	0.9575	0.9483	0.9548	0.9286	0.9335	0.9542	0.9535	0.9557	0.9466	0.9502
	TEB	0.9548	0.9489	0.9509	0.9349	0.9430	0.9607	0.9615	0.9590	0.9546	0.9623
	NB	0.9340	0.9422	0.9410	0.9439	0.9373	0.9505	0.9493	0.9471	0.9442	0.9525
7	TRB	0.9234	0.9556	0.9181	0.9405	0.9307	0.9375	0.9514	0.9394	0.9428	0.9461
	TEB	0.9089	0.9548	0.9458	0.9251	0.9438	0.9427	0.9532	0.9510	0.9352	0.9541
	NB	0.9340	0.9421	0.9413	0.9444	0.9377	0.9502	0.9493	0.9470	0.9443	0.9526
8	TRB	0.9275	0.9475	0.9505	0.9486	0.9494	0.9418	0.9495	0.9540	0.9502	0.9570
	TEB	0.9656	0.9716	0.9639	0.9633	0.9654	0.9594	0.9679	0.9659	0.9657	0.9698
	NB	0.9365	0.9448	0.9365	0.9435	0.9385	0.9516	0.9493	0.9460	0.9435	0.9514
9	TRB	0.9543	0.9533	0.9555	0.9385	0.9409	0.9559	0.9578	0.9584	0.9534	0.9567
	TEB	0.9617	0.9647	0.9553	0.9501	0.9473	0.9613	0.9647	0.9593	0.9594	0.9613
	NB	0.9342	0.9422	0.9411	0.9439	0.9376	0.9505	0.9492	0.9471	0.9442	0.9525

*Note*: Small and large sample sizes: $n_{S1}=12,n_{S2}=18,n_{S3}=10,n_{S4}=16,n_{S5}=14,n_{L1}=n_{L2}=100,n_{L3}=106,n_{L4}=110,n_{L5}=104.$

Abbreviations: NB, No Borrowing; TEB, Treatment Effect Borrowing; TRB, treatment response borrowing.

In scenario 7 where the true $\theta_{k}$ are highly heterogeneous across all subtrials and the true mean of the control arm varies across subgroups, all approaches produce coverage that is below the target (95%) in nearly all subtrials (Table 2). For instance, in subtrial 3, TEB gives coverage probability of nearly 3% higher than TRB when the overall sample size is small, but which is also closest to the target. Overall, TEB gives higher coverage probability in most scenarios when the sample size is large.

In Figures S1 and S5, we present graphical results of a comparison of the empirical standard error (EmpSE) from the Bayesian modelling approaches. We also present additional results on the subtrial‐wise and familywise error‐rate control for each of the three analysis approaches in Table S2. We notice that TRB has the smallest EmpSE when the sample size is small but as this gets larger, TEB has the smallest EmpSE. Furthermore, the error rates (FWER and type I error) from the borrowing strategies are smaller than those of the no borrowing strategy.

## 4 APPLICATION TO CASE STUDY DATA SETS

### 4.1 Data description

We apply the TRB, TEB and NB approaches to Bayesian analyses of two case study data sets. Currently, there are no published randomised basket trials with a continuous primary outcome, based on our assessment of recent systematic reviews, a search of clinicaltrials.gov, and an ongoing systematic review in our group. We instead identified trials with continuous primary outcomes whose design matches that of a randomised basket trial to utilise as case studies. The first case study data is drawn from a trial of 60 participants randomised between different structured exercise and diet interventions. Participants were first randomly assigned to one of three different exercise regimes, and within each exercise regime subgroup, they were further randomised to two dietary interventions. Although not labelled as a basket trial, this could be considered as a randomised basket trial with three subtrials defined by the exercise regime assigned. Each subtrial then comprises the 20 patients further randomised to either diet 1 or diet 2. Both diet 1 and diet 2 are similar across the subtrials. The post‐intervention clinical outcome of interest was the pulse rate, a continuous measurement was taken at either three or four timepoints from baseline for each participant. We analyse the pulse rate data from the end of the trial. This data is publicly available here (Repeated measures analysis with R. UCLA: Statistical Consulting Group, n.d.).

The second case study data set is drawn from a multi‐centre, randomised, double‐blind, placebo‐controlled, proof‐of‐concept study of iscalimab in patients with primary Sjogren's syndrome (Fisher et al., 2020). Iscalimab is a novel anti‐CD40 monoclonal antibody that targets the CD40–CD154‐mediated T cell–B cell interactions which have been shown to be responsible for aberrant lymphocyte activation in inflamed tissue (Fisher et al., 2020). The trial was conducted in 10 sites across five different countries, United Kingdom, Germany, Switzerland, Hungary, and United States, recruiting patients aged 18–75 years. This trial had two cohorts. In cohort 1, 12 patients were randomised to receive subcutaneous iscalimab (3 mg/kg) or placebo for 12 weeks, after which patients on placebo would receive iscalimab up to week 32 (end of the trial). Cohort 2 (with 32 patients) followed a similar design as cohort 1 except that a higher dose of iscalimab (10 mg/kg) was administered. As such, this could be viewed as a randomised basket trial with two subtrials. The primary outcome was the ESSDAI score, which has a maximum possible score of 123 points, with higher scores indicating more severe symptoms. The minimum clinically important improvement in ESSDAI has been published as three points. Our interest is the comparison of ESSDAI scores between iscalimab and placebo patients at week 12. For this trial, the process to obtain individual level patient data would have been (lasting several months), and we resorted to obtaining all relevant information on the distribution of ESSDAI scores for each treatment arm in the two subtrials (mean ESSDAI score for baseline and at each timepoint, range of scores, SDs) from the chief investigator and the published paper. Using this information, we simulate data for 24 patients in subtrial 1 and 64 patients in subtrial 2. Note that one key assumption in our analysis is that the difference in dosage of Iscalimab between the cohorts does not influence the outcomes. We view that as appropriate as our goal is to illustrate how different the results of the analyses can be, and what the differences may look like in a real trial.

### 4.2 Analysis of case studies

Table 3 presents the results of the three analyses (TRB, TEB, NB) of the two case study datasets. Our results demonstrate that TRB and TEB can give substantially different point estimates for the $\theta_{k}$. This is a consequence of the differential information borrowing from complementary subtrials.

**TABLE 3:** Bayesian analysis of the two case studies

		Subtrial		
Case study	Modelling strategy	1	2	3
Case study 1: All $n_{k}=20$	NB	2.50 (−2.11, 7.07)	4.0 (−2.83, 10.75)	23.59 (16.39, 30.01)
	TRB	1.73 (−2.81, 5.97)	6.27 (−0.29, 12.30)	27.45 (19.96, 34.98)
	TEB	1.31 (−0.12, 5.46)	2.23 (−3.63, 7.85)	33.27 (23.91, 48.71)
Case study 2: $n_{1}=24,n_{2}=64$	NB	0.21 (−2.26, 2.72)	−3.10 (−4.65, −1.46)	—
	TRB	−0.52 (−2.60, 1.55)	−2.73 (−4.18, −1.19)	—
	TEB	−0.46 (−2.61, 1.74)	−2.59 (−4.21, −0.82)	—

*Notes*: Data are the posterior mean treatment effects, $\theta_{k}$ (95% credible interval). NB refers to the analysis strategy without borrowing; TEB is where borrowing is performed based on the commensurability of the treatment effects $\theta_{k}$; TRB is where borrowing is performed based on the commensurability of the mean response across the respective treatment arms $\varphi_{\mathrm{jk}}$.

For case study 1, we notice that TEB has the smallest estimates in subtrials 1 and 2 and the largest in subtrial 3. Considering the posterior probabilities $\mathbb{P}\left(\theta_{k}>3|\text{data}\right)$, we see that for subtrial 2, $\mathbb{P}\left(\theta_{2}>3|\text{data}\right)=0.615,0.852$ and 0.40 for no borrowing, TRB and TEB respectively (see Table 4). Thus, if a typical threshold probability to inform decision‐making such as ζ=0.9 was specified (i.e., decision‐making was based on $\mathbb{P}\left(\theta_{k}>3|\text{data}\right)>\zeta$), we would arrive at identical *no‐go* conclusions for all subtrials since all the posterior probabilities $\mathbb{P}\left(\theta_{k}>3|\text{data}\right)$ are less than ζ=0.9. However, if a smaller value such as ζ=0.8 is chosen for some trials, for example as in the case of a small proof‐of‐concept study, we can see the TRB and TEB analyses would then lead to different conclusions in subtrial 2 of case study 1; there is an 85.2% chance of recommending the intervention by the TRB analysis, while the NB and TRB analyses, this probability is 61.5% and 40%, respectively.

**TABLE 4:** Posterior probability that $\theta_{k}$ exceeds a pre‐specified threshold, $\mathbb{P}\left(\theta_{k}>\vartheta |\text{data}\right)$

Case study	Modelling strategy	1	2	3
Case study 1: All $n_{k}=20$	NB	0.419	0.615	0.999
	TRB	0.275	0.852	1.000
	TEB	0.239	0.398	1.000
Case study 2: $n_{1}=24,n_{2}=64$	NB	0.016	0.247	—
	TRB	0.039	0.365	—
	TEB	0.028	0.468	—

*Note*: In case study 1, ϑ=5 and ϑ=3 for case study 2.

Abbreviations: NB, No Borrowing; TEB, Treatment Effect Borrowing; TRB, treatment response borrowing.

As in case study 1, we notice that the treatment effects for case study 2 are different between the NB approach and the two modelling strategies TEB and TRB (Table 3). Here TRB and TEB produce comparable results, both for the treatment effects and the 95% credible intervals, an observation more likely when the groupwise mean responses are comparable across subtrials. However, we notice that the posterior probabilities to inform go/no‐go decisions were slightly different (0.365 vs. 0.468) (Table 4). While no more than three subtrials are analysed here and our simulation study had five subtrials, there is no expectation for the number of subtrials to have an impact. In principle, the distributional discrepancy approach here allows for borrowing depending only on the commensurability of information across subtrials.

## 5 DISCUSSION

The use of basket trials has grown substantially in the last decade, especially in early‐phase trial settings targeting rare diseases (Hazim & Prasad, 2018; Park et al., 2019). Randomised basket trials provide higher‐quality evidence of treatment benefit over the control relative to non‐randomised basket trials and will increasingly characterise the future landscape of phase II and phase III basket trials given the enormous weight for randomised evidence in drug approvals (Prasad & Oseran, 2015; Saad et al., 2017). Some recent ongoing randomised basket studies include NCT04498962 and NCT04988087.

In this article, we sought to understand the best modelling strategy to borrow information in the Bayesian analysis of a randomised basket trial with a continuous endpoint, motivated in part by our desire to identify a suitable analysis approach for the OACS randomised basket trial. We have proposed a TRB approach focusing on the borrowing by treatment arms (groupwise responses) and compared its performance to TEB as well as the NB approach. Specifically, the TRB approach assesses how similar the responses to a specific treatment are across subtrials, in contrast to the TEB that assesses how similar the experimental treatment benefit over the control (treatment effects) is across subtrials, for leveraging information from commensurate subtrials.

Our work is relevant to debates on the arm‐based (Hong et al., 2016) against contrast‐based (Begg & Pilote, 1991) frameworks for performing meta‐analysis of randomised controlled trials with at least two treatment arms (Dias & Ades, 2016). The newly proposed TRB offers added flexibility to implementing borrowing of information within basket trials. Firstly, data from complementary subtrials that are commensurate with the contemporary subtrial by either the experimental or control arm can be leveraged. Secondly, incommensurate data can be down weighted to a different extent by treatment arms. Our results are congruent with studies comparing arm‐based and contrast‐based models; estimates from arm‐based models were slightly larger than those obtained from contrast‐based models, but with smaller SEs (Karahalios et al., 2022).

Both TRB and TEB are based on the commensurate predictive prior approach to quantifying the degree of commensurability; synthesis weights are specified as a function of the pairwise Hellinger distance and assigned to the corresponding complementary subtrial. When fitting the Bayesian models with a TRB or TEB approach, we placed a spike and slab prior on the precision parameter that gauges the degree of borrowing, but alternative priors could be considered. Zheng et al. used a two‐component gamma mixture prior instead for analytic tractability in the exact Bayesian inference of treatment effects when leveraging pre‐experimental data from multiple sources (Zheng et al., 2020). Using a two‐component gamma mixture prior brings about the convenience of analytic tractability and would particularly facilitate the derivation of a sample size formula for the trial design. The two choices of prior on the commensurate parameter would mean different interpretation/implication of a specified value for the prior probability of incommensurability. For instance, setting $\omega_{kk^{*}}$ to a value as small as 0.3 may suggest a priori strong borrowing in the models coupled with a spike and slab prior, while the degree of borrowing might be attenuated with a two‐component gamma mixture prior. Indeed, we expect differences associated with these two prior specifications, and it is our plan to perform another comprehensive simulation study to formally compare the marginal changes in the degree of borrowing induced by these two prior specifications.

Our simulation results show that permitting borrowing of information in the analysis of randomised basket trials offers substantial gains in efficiency over independent analysis of subtrials (NB) as shown in previous simulation studies (Jin et al., 2020; Zheng & Wason, 2022). The proposed approaches to TRB and TEB differ most widely in performance when subtrial sample sizes are small, with differences depending on the similarity of treatment effects or treatment responses within each subtrial. In circumstances of small subtrial sizes, TRB typically outperforms TEB, especially when either the treatment effects or the mean response in control (or both) are consistent across subtrials. On the contrary, TEB performs better when there is considerable heterogeneity in treatment effects and/or the mean response in either the control or experimental arm across subtrials. Our findings indicate that TEB is a more robust approach that provides more precise estimates with better coverage probability and marginally higher power over TRB in nearly all scenarios when the trial sample size is sufficiently large. We also applied both TRB and TEB to the analysis of two case study data sets, showing that a trial may arrive at a different conclusion based on the chosen analysis approach. An important note is that our choice of case study 1 is different from the common basket design (Figure 1). This helps illustrate that basket trials are equally applicable to non‐oncology settings and non‐pharmaceutical interventions and are not restricted to biomarker‐guided pharmaceutical interventions as is common in oncology.

While we have considered a single Bayesian analysis methodology in Section 2, the concept underlying TRB can be implemented using any proposed Bayesian hierarchical method for basket trials. For illustrative purposes, we have focused on a setting where patients are equally randomised to a single experimental treatment or control within each subtrial, with a common continuous endpoint. A possible extension to our proposed methodology is thus to consider binary or time to event outcomes like progression‐free survival. In a binary setting, we can assume response in each subgroup follows a binomial distribution, and we can model the log‐odds of response. Borrowing in this case would be based on the similarity of the difference in log‐odds per subgroup for TEB, while for TRB we account for the similarity of the log‐odds separately for control and treatment arms across subgroups. The log‐odds can be assumed to follow a normal distribution, with normal hyperpriors and the Bayesian hierarchical modelling proceeds as in Section 2.1 and 2.2.

There are also several avenues to enhance the practicality of our proposed methodology (particularly the TRB strategy) in precision medicine trials. One future direction is to explore its performance in a basket trial setting where patients are randomised to at least two experimental treatment groups plus control in all or some of the subtrials. Another type of randomised basket design could consider incorporating response‐adaptive randomization (Lin et al., 2021; Ventz et al., 2017); more specifically, the randomization ratio could be adapted at an interim analysis to favour a treatment with the highest efficacy, as estimated by our proposed TRB. Ventz and colleagues recently developed a class of Bayesian response adaptive basket designs and demonstrated the advantages of adapting randomization with borrowing over not borrowing (Ventz et al., 2017). Furthermore, it is also possible to incorporate early stopping for futility or efficacy. For example, we may decide to drop subgroup k if the posterior probability that the treatment effect $\theta_{k}$ exceeds a prespecified threshold $\vartheta_{0}$ is low, that is, $\mathrm{Pr}\left(\theta_{k}>\vartheta_{0}\right)\leq \delta$ for TRB. Similarly, if the posterior probability is high, the subgroup can be stopped for efficacy. An important note here is that the decision‐making criteria using $\theta_{k}$ is the same TRB or TEB as this decision is made post‐computation of the treatment effects.

The use of TRB is in no way restricted to basket trial designs. Our proposed methodology could potentially be used in umbrella trials with a common control in each subtrial. The Lung‐MAP umbrella trial (Ferrarotto et al., 2015) is one such example where this may be plausible. Here, information borrowing across subtrials would be implemented for the control arm only. Relevant to this context, Zang et al. proposed a Bayesian hierarchical model to borrow information across subgroups receiving standard‐of‐care only in a Bayesian adaptive marker‐stratified design (Zang et al., 2019). By using the TRB proposed in this paper, we could expect a more precise estimate of the mean response in each control arm to be achieved, which would aid the estimation of the treatment effect specific to each subgroup.

We caution that despite the efficiencies of borrowing across subgroups in basket trials, this should always be motivated by a strong biological and clinical rationale. For instance, when the control (e.g., the standard of care treatment) is different in certain subgroups, the strength of scientific and clinical evidence underpinning such borrowing is weak. This is because different drugs may likely have potentially different mechanisms of action and may elicit different responses across subgroups. As such, borrowing is more justifiable only across similar controls and TRB would be suitable if the experimental arm is the same, but the control arm is not. We also note that Bayesian simulations undertaken here demand high computational requirements. The computational time for a single scenario with 10,000 simulation replicates, two MCMC chains each with 10,000 iterations for TEB/TRB was 3.four to five hours on average when parallelising on 44 cores in a high‐performance computing cluster node (2.2 GHz Intel Xeon E5‐2699 v4 processor). For the NB approach, this takes approximately 10 min.

Finally, we conclude that borrowing of information in randomised basket trials offers considerable efficiency gains but must be implemented carefully depending on the potential heterogeneity in effects across subgroups and the subtrial sample sizes. Consequently, investigators and statisticians must thoroughly investigate any borrowing approach based on plausible trial scenarios.

## CONFLICT OF INTEREST

None to be declared.

## Supporting information


**Figure S1:** Comparison of the Empirical standard error (EmpSE).
**Table S1:** Comparison of the coverage probability for the 95% posterior credible interval.
**Figure S2:** A comparison of the frequentist power based on the borrowing of information approaches in a randomised basket trial.
**Table S2:** Comparison of the frequentist type I error‐rate and familywise error‐rate (FWER) based on the different borrowing of information strategies in a randomised basket trial.
**Figure S3:** Comparison of three Bayesian analysis models for randomised basket trials, in terms of the bias and mean squared error of the estimators under varying sample sizes.
**Figure S4:** A comparison of the Bayesian analysis models for randomised basket trials, in terms of the frequentist power. Frequentist power is defined as the proportion of trials with a correct *go* decision under $H_{1k}$.
**Figure S5:** Comparison of the empirical standard error (EmpSE) under varying sample sizes.
**Table S3:** Posterior probability that $\theta_{k}$ exceeds a pre‐specified threshold, ℙ(θ>δ|data). In case study 1, δ=5 and δ=3 for case study 2.
**Figure S6:** Comparison of the median width of the 95% credible interval for posterior estimates $\theta_{k}$ obtained from different Bayesian analysis models under varying sample sizes. Error bars represent the 10th and 90th percentiles.

## Data Availability

Case study data alongside relevant analysis code are available at https://github.com/oondijo/RandBasketTrials.
