# Supplementary material for: Bayesian modelling strategies for borrowing of information in randomised basket trials
Source: J R Stat Soc Ser C Appl Stat. 2022 Oct 28;71(5):2014–37. doi: 10.1111/rssc.12602 (PMC9827857; doi:10.1111/rssc.12602)
Supplement: Supplementary file 1 — Figure S1: Comparison of the Empirical standard error (EmpSE). Table S1: Comparison of the coverage probability for the 95% posterior credible interval. Figure S2: A comparison of the frequentist power based on the borrowing of information approaches in a randomised basket trial. Table S2: Comparison of the frequentist type I error‐rate and familywise error‐rate (FWER) based on the different borrowing of information strategies in a randomised basket trial. Figure S3: Comparison of three Bayesian analysis models for randomised basket trials, in terms of the bias and mean squared error of the estimators under varying sample sizes. Figure S4: A comparison of the Bayesian analysis models for randomised basket trials, in terms of the frequentist power. Frequentist power is defined as the proportion of trials with a correct go decision under H1k. Figure S5: Comparison of the empirical standard error (EmpSE) under varying sample sizes. Table S3: Posterior probability that θk exceeds a pre‐specified threshold, ℙ(θ>δ|data). In case study 1, δ=5 and δ=3 for case study 2. Figure S6: Comparison of the median width of the 95% credible interval for posterior estimates θk obtained from different Bayesian analysis models under varying sample sizes. Error bars represent the 10th and 90th percentiles. [file RSSC-71-2014-s001.docx]

Web based supporting materials for: **Bayesian modelling strategies for borrowing of information in randomised basket trials**

by Luke O. Ouma, Michael J. Grayling, James M.S. Wason, Haiyan Zheng

## Supplementary methods

**Treatment response borrowing**

Suppose we are interested in estimating $\varphi_{jk}$ (when $(K\geq3)$, we can represent the $K-1$ CPPs marginally on $\varphi_{jk}$ as follows

| $\int\pi^{cpp}\left( \varphi_{jk},\nu_{kk^{*}}^{\left( j \right)}\vert\boldsymbol{x}_{k}^{\left( j \right)},\varphi_{{jk}^{*}} \right)d\nu_{kk^{*}}^{\left( j \right)}\propto$  $\int L\left( \boldsymbol{x}_{k^{*}}^{\left( j \right)}\vert\varphi_{{jk}^{*}} \right)\pi_{0k^{*}}\left( \varphi_{{jk}^{*}} \right)\times\nu_{kk^{*}}^{\left( j \right)}\phi\left( \left( {\varphi_{jk}-\varphi}_{{jk}^{*}} \right)\nu_{kk^{*}}^{\left( j \right)} \right)g\left( \nu_{kk^{*}}^{\left( j \right)} \right)d\nu_{kk^{*}}^{\left( j \right)},$ | (1) |
| --- | --- |

where $\phi$ is the standard normal density function and values of $\nu_{kk^{*}}^{\left( j \right)}$ are appropriately specified.

**Treatment effect borrowing**

Suppose we are interested in estimating $\theta_{k}$ (when $K=2)$, conditional on information commensurability, we have the commensurate predictive prior

| $\pi^{cpp}\left( \theta_{k},v_{kk^{*}}\vert x_{k^{*}},\theta_{k^{*}} \right)\propto$  $L\left( \theta_{k^{*}}\vert x_{k^{*}} \right)\times\pi_{0k^{*}}\left( \theta_{k^{*}} \right)\times v_{kk^{*}}\phi\left( \left( \theta_{k}-\theta_{k^{*}} \right)v_{kk^{*}} \right)\times g\left( v_{kk^{*}} \right)$ | (2) |
| --- | --- |

Suppose we are interested in estimating $\theta_{k}$ (when$K\geq3$), we can represent the $K-1$ CPPs marginally on $\theta_{k}$ as follows

| $\int\pi^{cpp}\left( \theta_{k},v_{kk^{*}}\vert\boldsymbol{x}_{k},\theta_{k^{*}} \right)dv_{kk^{*}} \propto$  $\int L\left( \boldsymbol{x}_{k^{*}}\vert\theta_{k^{*}} \right)\pi_{0k^{*}}\left( \theta_{k^{*}} \right)\times v_{kk^{*}}\phi\left( \left( {\theta_{k}-\theta}_{k^{*}} \right)v_{kk^{*}} \right)g\left( v_{kk^{*}} \right)dv_{kk^{*}}$ | (3) |
| --- | --- |

where $\phi$ is the standard normal density function and $v_{kk^{*}}$ is appropriately specified.

## Supplementary results

### Other performance measures


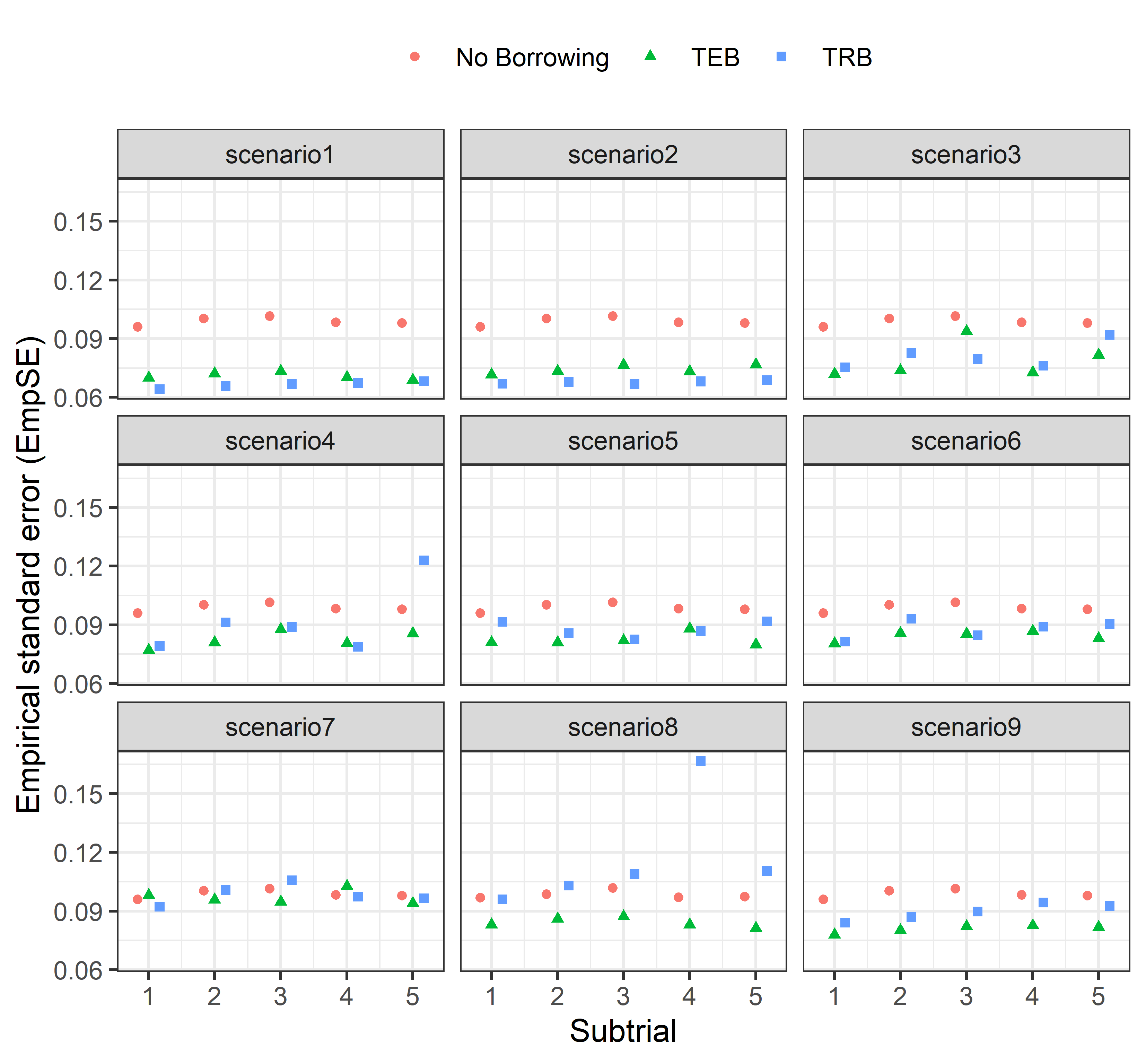


**Supplementary Figure 1:** Comparison of the Empirical standard error (EmpSE).


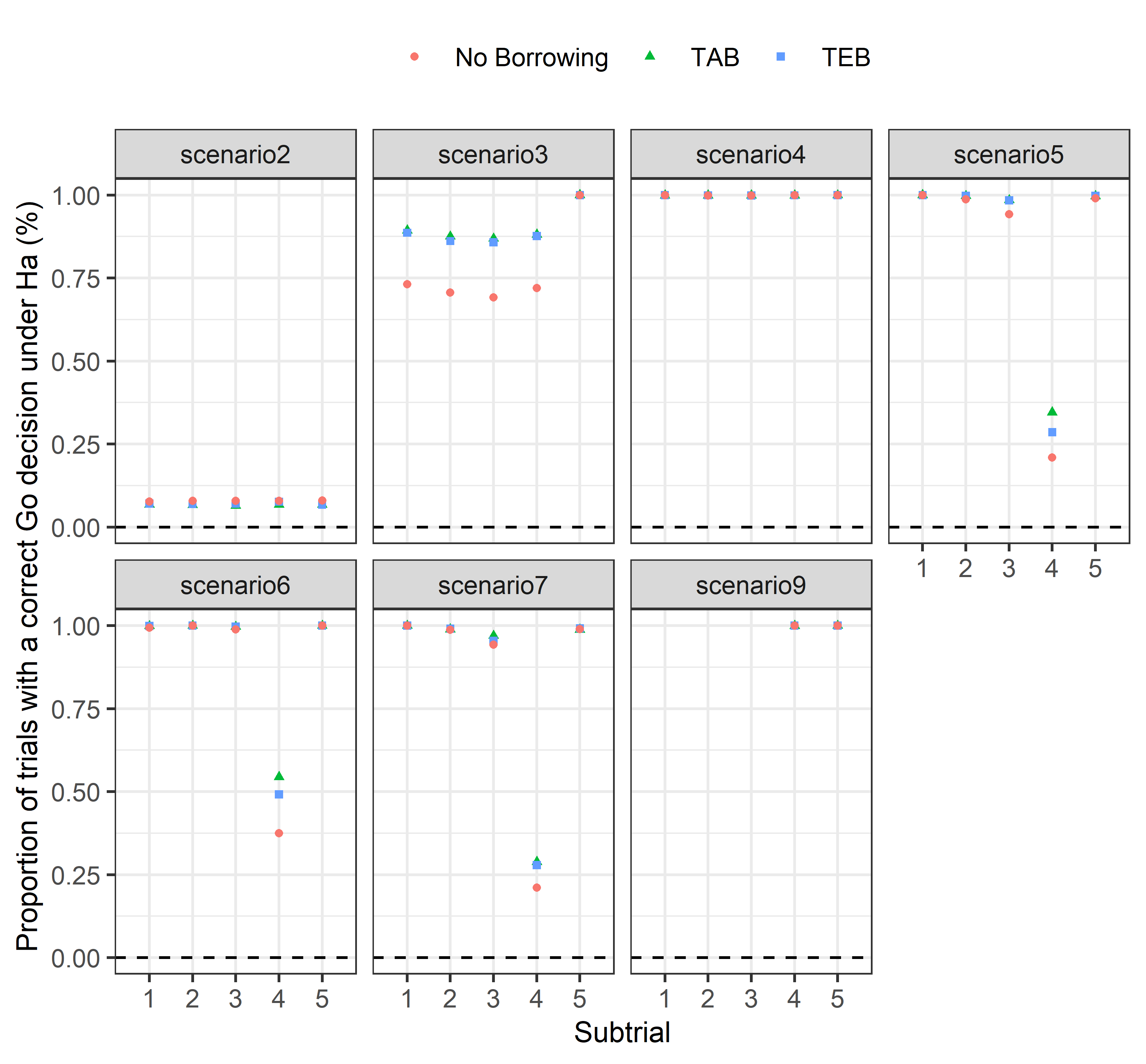


**Supplementary Figure 2:** A comparison of the frequentist power based on the borrowing of information approaches in a randomised basket trial.

**Supplementary Table 1:** Comparison of the coverage probability for the 95% posterior credible interval.

| Scenario | Modelling strategy | Coverage probability | | | | |
| --- | --- | --- | --- | --- | --- | --- |
|  |  | S1 | S2 | S3 | S4 | S5 |
| Scenario 1 | TRB | 0.9793 | 0.9774 | 0.9775 | 0.9774 | 0.9765 |
|  | TEB | 0.9765 | 0.9746 | 0.9750 | 0.9756 | 0.9769 |
|  | NB | 0.9461 | 0.9424 | 0.9457 | 0.9473 | 0.9446 |
| Scenario 2 | TRB | 0.9794 | 0.9770 | 0.9778 | 0.9784 | 0.9770 |
|  | TEB | 0.9733 | 0.9704 | 0.9687 | 0.9701 | 0.9670 |
|  | NB | 0.9462 | 0.9423 | 0.9457 | 0.9474 | 0.9446 |
| Scenario 3 | TRB | 0.9734 | 0.9684 | 0.9706 | 0.9685 | 0.9513 |
|  | TEB | 0.9749 | 0.9701 | 0.9691 | 0.9709 | 0.9600 |
|  | NB | 0.9462 | 0.9423 | 0.9457 | 0.9474 | 0.9446 |
| Scenario 4 | TRB | 0.9743 | 0.9694 | 0.9717 | 0.9696 | 0.9593 |
|  | TEB | 0.9715 | 0.9676 | 0.9693 | 0.9689 | 0.9651 |
|  | NB | 0.9462 | 0.9424 | 0.9457 | 0.9474 | 0.9445 |
| Scenario 5 | TRB | 0.9481 | 0.9626 | 0.9641 | 0.9371 | 0.9633 |
|  | TEB | 0.9614 | 0.9606 | 0.9620 | 0.9482 | 0.9636 |
|  | NB | 0.9461 | 0.9424 | 0.9457 | 0.9474 | 0.9446 |
| Scenario 6 | TRB | 0.9641 | 0.9465 | 0.9605 | 0.9435 | 0.9469 |
|  | TEB | 0.9637 | 0.9544 | 0.9602 | 0.9518 | 0.9599 |
|  | NB | 0.9462 | 0.9424 | 0.9457 | 0.9474 | 0.9446 |
| Scenario 7 | TRB | 0.9428 | 0.9482 | 0.9385 | 0.9358 | 0.9431 |
|  | TEB | 0.9453 | 0.9485 | 0.9526 | 0.9308 | 0.9523 |
|  | NB | 0.9462 | 0.9424 | 0.9456 | 0.9472 | 0.9447 |
| Scenario 8 | TRB | 0.9487 | 0.9438 | 0.9560 | 0.9491 | 0.9588 |
|  | TEB | 0.9675 | 0.9652 | 0.9689 | 0.9682 | 0.9743 |
|  | NB | 0.9453 | 0.9473 | 0.9460 | 0.9496 | 0.9481 |
| Scenario 9 | TRB | 0.9635 | 0.9592 | 0.9610 | 0.9563 | 0.9555 |
|  | TEB | 0.9667 | 0.9650 | 0.9620 | 0.9593 | 0.9610 |
|  | NB | 0.9461 | 0.9424 | 0.9457 | 0.9474 | 0.9447 |

Note: S1-S5 denote the subtrials

**Supplementary Table 2:** Comparison of the frequentist type I error-rate and familywise error-rate (FWER) based on the different borrowing of information strategies in a randomised basket trial.

| Scenario | Modelling Strategy | Type I error | | | | | |  |
| --- | --- | --- | --- | --- | --- | --- | --- | --- |
|  |  | S1 | S2 | S3 | S4 | S5 | FWER | |
| Scenario 1 | TRB | 0.0000 | 0.0000 | 0.0002 | 0.0000 | 0.0001 | 0.0003 | |
|  | TEB | 0.0000 | 0.0000 | 0.0005 | 0.0000 | 0.0002 | 0.0007 | |
|  | NB | 0.0014 | 0.0000 | 0.0012 | 0.0005 | 0.0009 | 0.0040 | |
| Scenario 8 | TRB | 0.0024 | 0.0003 | 0.0008 | 0.0001 | 0.0001 | 0.0037 | |
|  | TEB | 0.0001 | 0.0002 | 0.0010 | 0.0000 | 0.0004 | 0.0017 | |
|  | NB | 0.0012 | 0.0002 | 0.0015 | 0.0005 | 0.0007 | 0.0041 | |

Note: S1-S5 denote the subtrials

### Performance measures under different sample sizes


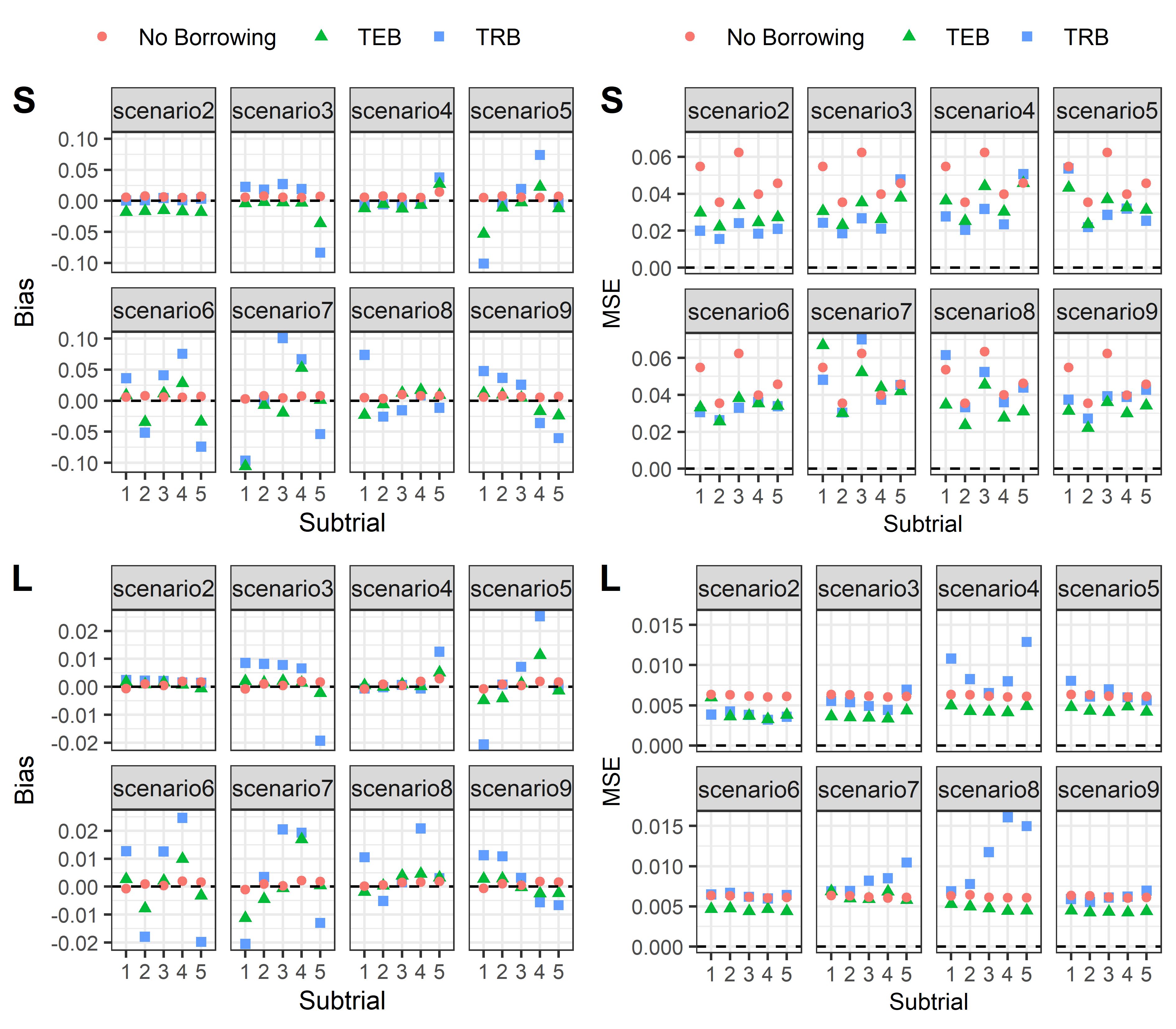


**Supplementary Figure 3:** Comparison of three Bayesian analysis models for randomised basket trials, in terms of the bias and mean squared error of the estimators under varying sample sizes.


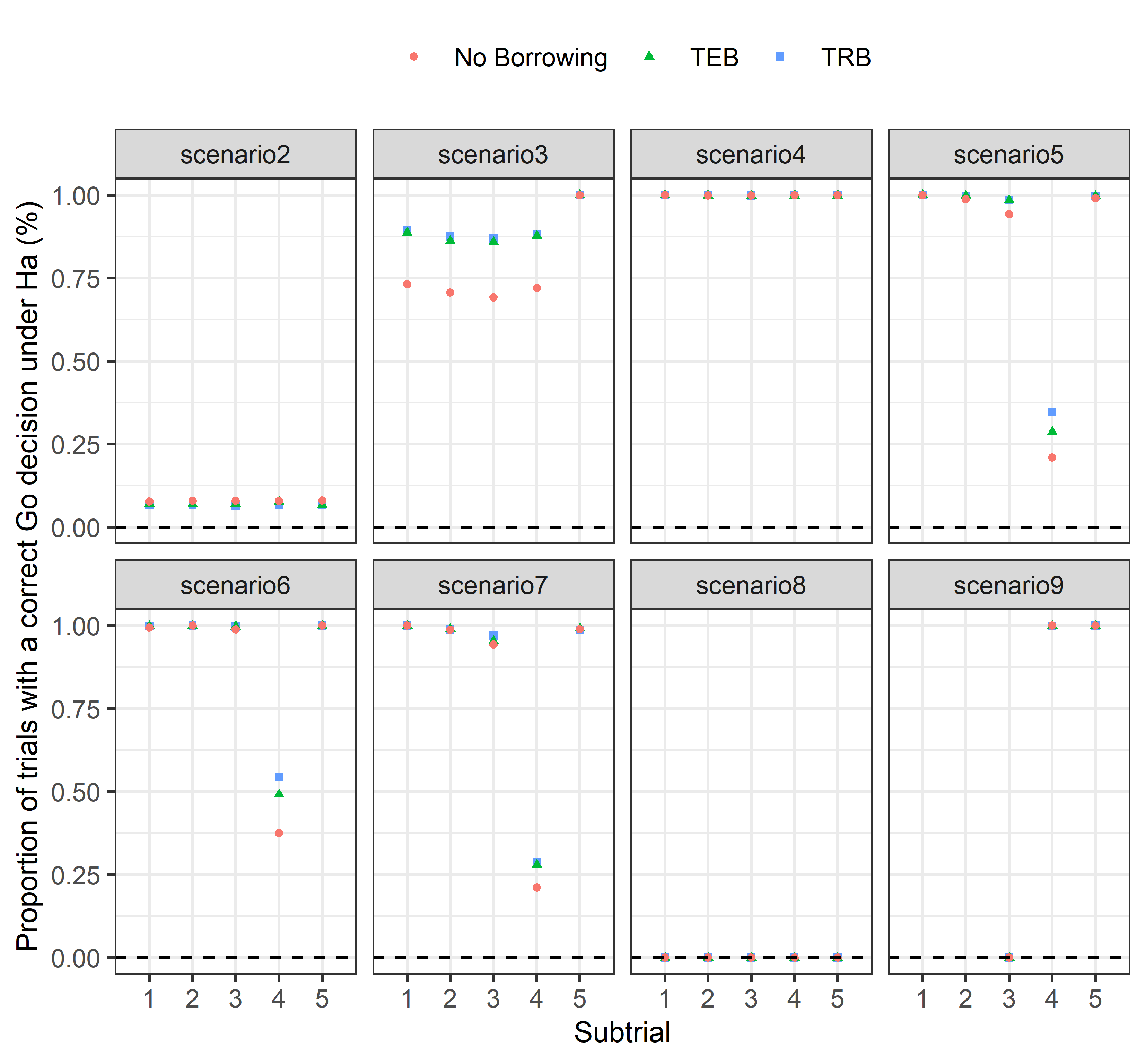


**Supplementary Figure 4:** A comparison of the Bayesian analysis models for randomised basket trials, in terms of the frequentist power. Frequentist power is defined as the proportion of trials with a correct *go* decision under $H_{1k}$.


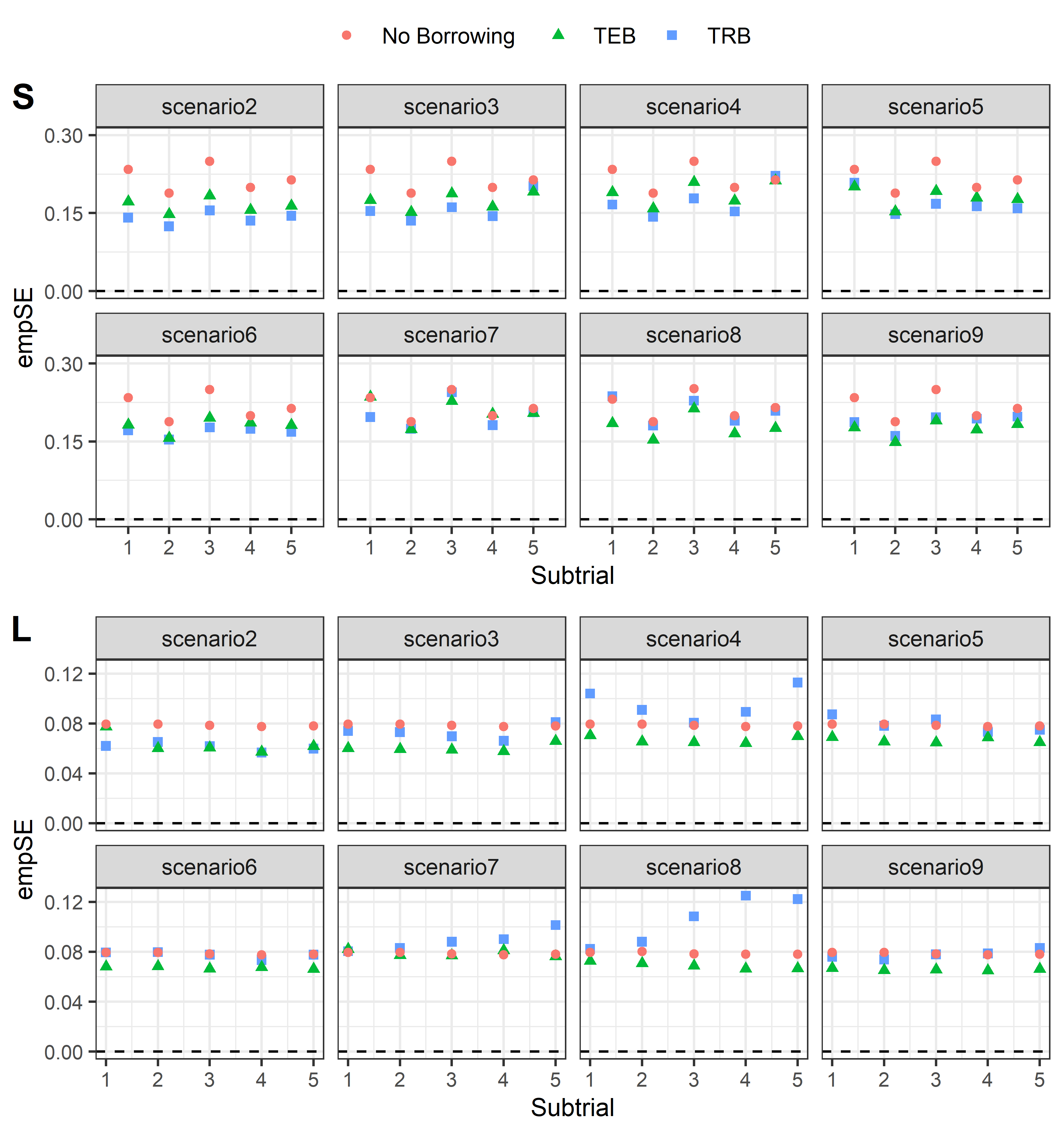


**Supplementary Figure 5:** Comparison of the empirical standard error (EmpSE) under varying sample sizes.


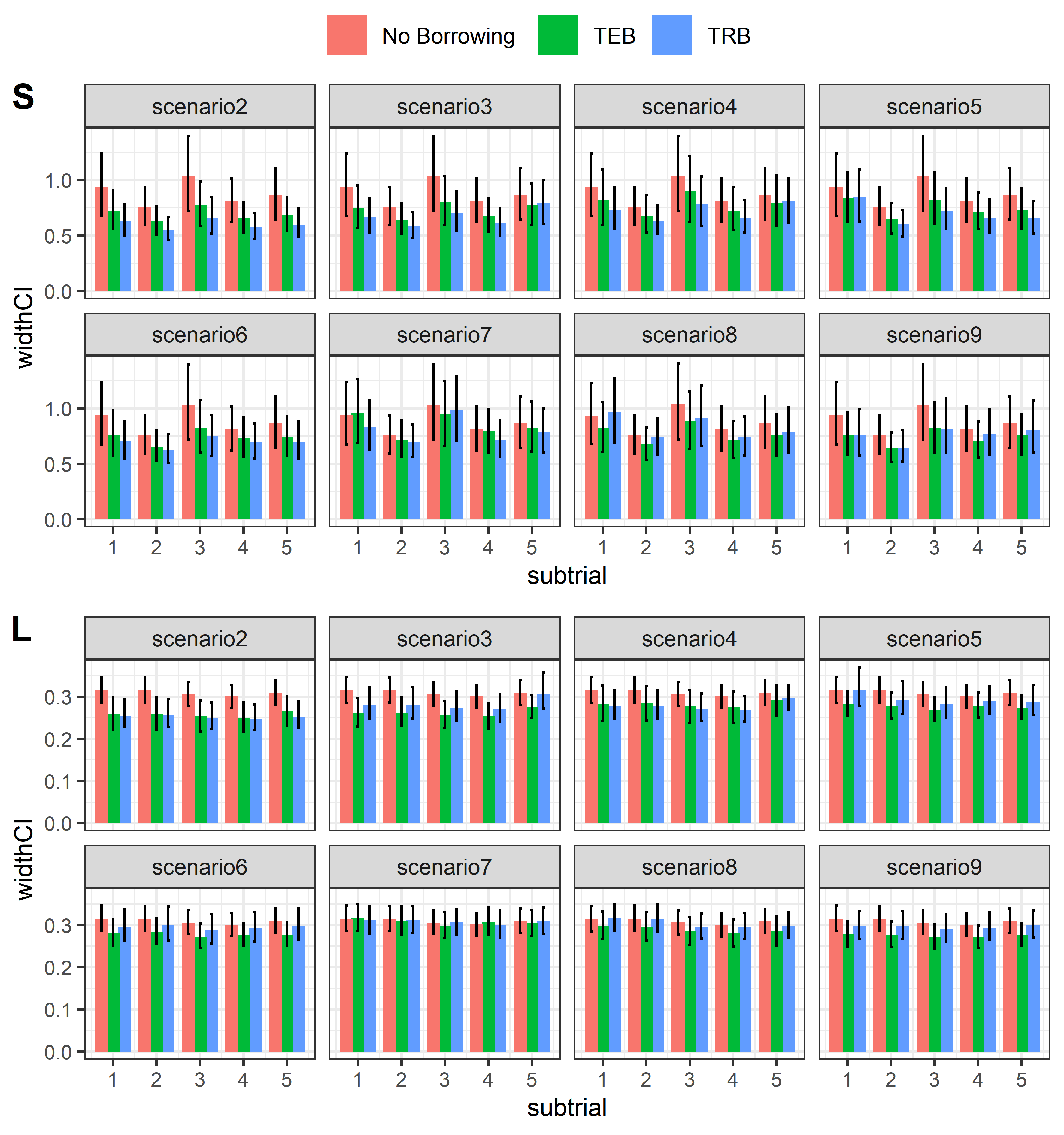


**Supplementary Figure 6:** Comparison of the median width of the 95% credible interval for posterior estimates $\theta_{k}$ obtained from different Bayesian analysis models under varying sample sizes. Error bars represent the 10^th^ and 90^th^ percentiles.

**Supplementary Table 3:** Posterior probability that $\theta_{k}$ exceeds a pre-specified threshold, $\mathbb{P}(\theta>\delta|data)$. In case study 1, $\delta=5$ and $\delta=3$ for case study 2.

| Case study | Modelling strategy | 1 | 2 | 3 |
| --- | --- | --- | --- | --- |
| Case study 1  All $n_{k}=20$ | NB | 0.4185 | 0.6145 | 0.999 |
|  | TRB | 0.2750 | 0.8515 | 1 |
|  | TEB | 0.2390 | 0.3975 | 1 |
| Case study 2  $n_{1}=24, n_{2}=64$ | NB | 0.016 | 0.2465 | - |
|  | TRB | 0.039 | 0.365 | - |
|  | TEB | 0.0275 | 0.468 | - |
